# Efficacy and safety of Buyang Huanwu Decoction combined with α-lipoic acid for diabetic peripheral neuropathy: a systematic review with in-depth heterogeneity deconstruction and methodological appraisal

**DOI:** 10.3389/fendo.2026.1778262

**Published:** 2026-04-30

**Authors:** Qi Yong, Peiying Zhang, Yunxi Xu, Chao Xu, Hua Bai, Hejiang Ye

**Affiliations:** 1 Clinical Medical College, Chengdu University of Traditional Chinese Medicine, Chengdu, Sichuan, China; 2 Department of Ophthalmology, Hospital of Chengdu University of Traditional Chinese Medicine, Chengdu, Sichuan, China

**Keywords:** Buyang Huanwu Decoction, diabetic peripheral neuropathy, heterogeneity, meta-analysis, nerve conduction velocity, TCM syndrome, α-lipoic acid

## Abstract

**Background:**

Diabetic peripheral neuropathy (DPN) is a common diabetes complication. Buyang Huanwu Decoction (BYHWD) combined with α-lipoic acid (ALA) is used clinically, but evidence consistency and heterogeneity sources remain unexplored.

**Objective:**

To evaluate efficacy/safety of BYHWD+ALA for DPN and deconstruct heterogeneity via a multi−tiered framework.

**Methods:**

We searched PubMed, Web of Science, EMbase, Cochrane Library, CNKI, Wanfang, VIP, and CBM up to December 2025 for RCTs. Two reviewers independently screened studies, extracted data, and assessed risk of bias (Cochrane tool). Meta−analysis used RR (dichotomous) and SMD (continuous) with 95% CI. Heterogeneity (I²) was explored through subgroup analysis, sensitivity analysis, meta−regression, and descriptive synthesis.

**Results:**

Twelve RCTs (926 patients) were included. BYHWD+ALA significantly improved overall response rate (RR = 1.24, 95%CI 1.17–1.32; I²=0%) and TCM syndrome scores (SMD=–0.76, 95%CI –0.98 to –0.54). For nerve conduction velocity (NCV), fundamental heterogeneity emerged (I²>92%). A five−tiered analysis revealed that only peroneal nerve motor NCV showed a robust improvement trend (SMD = 1.01, 95%CI 0.48–1.54), though heterogeneity remained high (I²=92%). Heterogeneity sources included non−standardized NCV measurement and complex intervention−patient matching. Oxidative stress markers (SOD, MDA, T−AOC) trended favorably; HbA1c and adverse events did not differ between groups.

**Conclusion:**

BYHWD+ALA safely improves subjective DPN symptoms, but NCV evidence is too heterogeneous to draw reliable quantitative conclusions. This study provides a methodological framework for deconstructing heterogeneity and guides future standardized research.

**Systematic Review Registration:**

https://www.crd.york.ac.uk/PROSPERO/display_record.php?RecordID=1267606, identifier CRD420251267606.

## 1 Introduction

Diabetic peripheral neuropathy (DPN) is the most prevalent and disabling chronic microvascular complication of diabetes (1). Recent epidemiological studies indicate that DPN affects approximately 50% of the global diabetic population, with its prevalence increasing significantly with diabetes duration, exceeding 50% in those with a disease course over 10 years (1). This poses a formidable public health challenge, leading to severely diminished quality of life, increased risk of foot ulcers and amputations, and a substantial socioeconomic burden (1, 32). Clinical manifestations include limb numbness, pain, paresthesia, and muscle weakness, which can progress to foot ulcers, infections, and even amputation in severe cases. The pathogenesis of DPN involves multifactorial processes including oxidative stress and microcirculatory impairment. Key processes include long-term hyperglycemia-induced oxidative stress, accumulation of advanced glycation end-products, polyol pathway activation, impaired neural blood supply, mitochondrial dysfunction, as well as emerging factors like neuroinflammation and lipid metabolism disorders. These elements collectively contribute to neuronal and Schwann cell injury, axonal degeneration, and demyelination (3). Notably, recent research has broadened the perspective, recognizing that hyperglycemia is not the sole driver. Obesity and dyslipidemia, particularly the accumulation of free fatty acids, have been identified as independent and critical risk factors for the development and progression of DPN, especially in type 2 diabetes. This has shifted the therapeutic paradigm from intensive glucose control alone to a comprehensive, multi-targeted management approach addressing oxidative stress, metabolic disturbances, and microcirculatory impairment (4).

Current clinical management strategies for DPN remain suboptimal, particularly lacking disease-modifying therapies capable of reversing structural nerve damage. Strict glycemic control is the cornerstone for delaying progression but offers limited improvement for established neuropathic symptoms (5). Authoritative international guidelines, such as the American Diabetes Association (ADA) position statement, emphasize that modern DPN management requires a multidisciplinary, stratified, and comprehensive strategy. Core components include early screening (detailed history and simple clinical examinations), accurate diagnosis (excluding other etiologies), and symptom-based individualized treatment. Among numerous pharmacological agents targeting pathogenic mechanisms, α-lipoic acid (ALA), a potent endogenous antioxidant, holds a unique position. Its amphiphilic nature allows it to efficiently scavenge various free radicals through direct and indirect pathways, regenerate endogenous antioxidants (glutathione, vitamins C/E), and improve neural microcirculation. Consequently, it is recommended by several national guidelines for alleviating DPN symptoms (6). Its core mechanism involves penetrating cells, particularly mitochondria, to neutralize excessive reactive oxygen species (ROS) generated during energy metabolism—a process considered central to oxidative damage in DPN (7). A recent meta-analysis on oral ALA confirmed its definite advantage in improving Total Symptom Score (TSS) in patients, also suggesting a dose-dependent effect. However, the same analysis noted inconsistent effects on objective electrophysiological indicators like nerve conduction velocity (NCV), implying a potential dissociation between functional improvement and structural repair (2). Another randomized controlled trial further confirmed that ALA effectively reduces vibration perception threshold (VPT) and neuropathy symptom scores, with its core mechanism closely linked to significantly lowering serum levels of oxidative stress and inflammatory biomarkers such as malondialdehyde (MDA) and high-sensitivity C-reactive protein (hs-CRP) (8). Nevertheless, the response rate and degree of symptom relief with monotherapy remain unsatisfactory, and long-term evidence for structural nerve repair needs strengthening, prompting exploration of synergistic combination therapies (9).

Traditional Chinese Medicine (TCM) has accumulated rich experience in the long-term clinical practice of managing DPN, forming a unique theoretical system. In TCM theory, DPN falls under the categories of “Bi Zheng” (impediment syndrome) or “Wei Zheng” (flaccidity syndrome) secondary to “Xiao Ke” (wasting-thirst, akin to diabetes). Its core pathogenesis is often attributed to “chronic illness consuming qi, leading to qi deficiency and blood stasis, resulting in collateral obstruction” (10). Qi deficiency impairs its propelling force, causing sluggish blood flow and subsequent stasis; blood stasis obstructs the collaterals, preventing qi and blood from nourishing the limbs, muscles, tendons, and vessels, thus manifesting as numbness, pain, and weakness. This pathogenic understanding highlights the dialectical relationship between “deficiency” (qi) and “excess” (stasis), providing a theoretical basis for treating DPN with the method of “tonifying qi and activating blood circulation” (11). Buyang Huanwu Decoction (BYHWD), originating from Wang Qingren’s “Yi Lin Gai Cuo” (Corrections of Errors in Medical Classics) from the Qing Dynasty, is a classic formula for tonifying qi, activating blood, and unblocking collaterals. The formula features a large dose of Astragalus membranaceus (Huang Qi, commonly 30-120g) as the sovereign herb to greatly supplement spleen and stomach qi, promoting blood circulation through vigorous qi. It is assisted by Angelica sinensis (Dang Gui Wei), Paeonia lactiflora (Chi Shao), Ligusticum chuanxiong (Chuan Xiong), Prunus persica (Tao Ren), and Carthamus tinctorius (Hong Hua) to activate blood and dispel stasis; Pheretima (Di Long) unblocks channels and collaterals. Combined, these herbs work complementarily to tonify qi, activate blood, resolve stasis, and unblock collaterals. Modern pharmacological research confirms that BYHWD and its active components possess multiple effects, including antioxidant, anti-inflammatory, microcirculation improvement, neuroprotective and Schwann cell protective properties, and regulation of autophagy and apoptosis (12). Recent basic research has further elucidated its deeper mechanisms, such as activating the AMPK/ULK1 pathway to enhance mitophagy and clear dysfunctional mitochondria, while inhibiting NLRP3 inflammasome-mediated pyroptosis, thereby exerting neuroprotection at the levels of cellular energy metabolism and programmed cell death (13). These multi-target, multi-level mechanisms exhibit significant synergistic and complementary potential with the antioxidant-centered action of ALA. In recent years, the number of clinical studies on TCM for DPN has grown significantly. An evidence mapping analysis covering the past decade indicates that integrated Chinese and Western medicine therapies (particularly Chinese herbal decoctions combined with conventional Western treatment) have become the most prevalent intervention model, showing potential in overall response rates and NCV improvement. Furthermore, a network meta-analysis suggested that among various Chinese herbal formulas combined with Western medicine, BYHWD ranked first in improving the clinical overall response rate for DPN (14, 38). However, research in this field also faces notable bottlenecks: most RCTs have small sample sizes, short intervention durations, and uneven methodological quality (e.g., inadequate reporting of randomization and blinding). Systematic reviews/meta-analyses themselves are often limited by high heterogeneity in primary studies, leading to fragmented evidence and uncertain conclusions (3, 33).

Several clinical studies in recent years have explored the efficacy and safety of BYHWD combined with ALA for DPN, preliminarily showing a promising “1 + 1>2” effect. However, these studies are mostly single-center, small-sample RCTs with significant variations in outcome selection (especially regarding the specific nerves and measurement sites for neuroelectrophysiological assessment), standardization of measurement methods, and reporting of TCM intervention details (e.g., Astragalus dose, herb origin, decoction method). This has resulted in inconsistent conclusions and severely fragmented evidence, hindering the formation of high-level clinical recommendations. Currently, there is not only a lack of systematic reviews on this specific combination regimen but also a paucity of studies employing systematic methods to deconstruct the root causes of evidence heterogeneity. Therefore, this study aims, through an innovative five-tiered progressive analytical framework, to not only quantitatively synthesize efficacy evidence but also deeply deconstruct the sources of heterogeneity, addressing deeper questions such as ‘why are the study results inconsistent?’ and ‘how should more rigorous studies be designed in the future?’. Consequently, this study aims to use systematic review and meta-analysis methods to quantitatively synthesize existing evidence and delineate the efficacy and safety profile of this combination regimen. More importantly, we will adopt a multi-level analytical strategy to systematically deconstruct the heterogeneity within the evidence body, revealing whether it stems from measurement issues, intervention details, or patient population characteristics. This approach seeks not only to answer “how effective is it?” but also to delve into “why are the study results inconsistent?” and “how should more rigorous studies be designed?”, providing deep-level evidence-based medical rationale and methodological insights for clinical decision-making and the construction of a high-quality, evaluable efficacy evidence system in this field.

## 2 Methods

### 2.1 Inclusion and exclusion criteria

Inclusion Criteria: (1) Study design: Randomized controlled trials (RCTs), regardless of blinding; (2) Participants: Adult patients diagnosed with DPN according to internationally or nationally recognized criteria; (3) Interventions: The experimental group received Buyang Huanwu Decoction (or modified) combined with α-lipoic acid and basic treatment; the control group received α-lipoic acid and basic treatment; (4) Outcome measures: Reported at least one of the following: overall response rate, TCM syndrome scores, nerve conduction velocity (NCV), oxidative stress markers (SOD, MDA, T-AOC), glycemic control indicator (HbA1c), Toronto Clinical Scoring System (TCSS) score, incidence of adverse events.

Exclusion Criteria: Non-RCT designs; incomplete data preventing extraction; intervention confounded by other Chinese herbal compound formulas; participants with severe hepatic/renal insufficiency or other serious primary diseases.

### 2.2 Literature search strategy

Computerized systematic searches were conducted in eight databases: PubMed, Web of Science, EMbase, Cochrane Library, China National Knowledge Infrastructure (CNKI), Wanfang Data, VIP Chinese Journal Database, and China Biology Medicine disc (CBM), as well as clinical trial registries (Chinese Clinical Trial Registry, ClinicalTrials.gov). The search timeframe spanned from database inception to December 2025. Chinese search terms included: “补阳还五汤”, “补阳还五”, “糖尿病周围神经病变”, “糖尿病神经病变”, “α-硫辛酸”, “硫辛酸”. English search terms included: “Buyang Huanwu Decoction”, “alpha-lipoic acid”, “Diabetic Neuropathies”, “Diabetic Peripheral Neuropathy”, “Randomized Controlled Trial”. Searches employed a combination of subject headings and free-text terms, adjusted for each database. The detailed search strategies are provided in **Supplementary Material S2**. References of included studies were also manually searched to identify additional relevant literature.

### 2.3 Study selection and data extraction

Search results were imported into EndNote X9 software to create a database. Two investigators (YQ and ZPY) independently performed study selection and data extraction. Discrepancies were resolved through discussion or consultation with a third investigator (XYX). Titles and abstracts were screened initially to exclude obviously irrelevant records. Full texts of the remaining records were then retrieved for further screening to finalize included studies. A pre-designed data extraction form was used to collect information, including: first author, publication year, study design, sample size, patient baseline characteristics (age, gender, duration of diabetes and DPN, staging), details of interventions (drug, dosage, administration, duration), control measures, and outcome data (means, standard deviations).

### 2.4 Methodological quality assessment

The methodological quality of the included RCTs was assessed using the Cochrane Collaboration’s “Risk of Bias” tool (version 1.0). Two investigators (YQ and ZPY) independently performed the assessment. Disagreements were resolved by consensus or arbitration by a third investigator (XYX). The tool evaluates seven domains, judging each as “low risk”, “high risk”, or “unclear risk”:

1. Random sequence generation;
2. Allocation concealment;
3. Blinding of participants and personnel;
4. Blinding of outcome assessment;
5. Incomplete outcome data;
6. Selective outcome reporting;
7. Other potential sources of bias.

### 2.5 Statistical analysis

**Figure 1:**
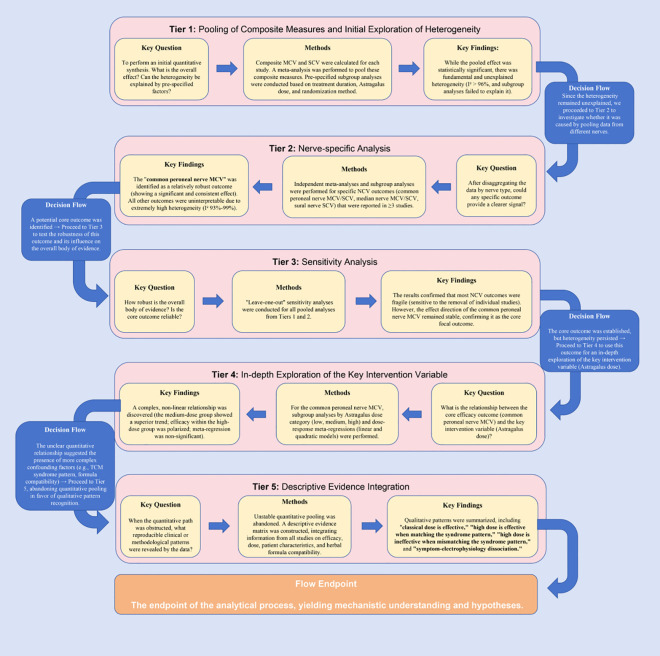
Flowchart of the five-tiered progressive analytical strategy for nerve conduction velocity evidence.

Meta-analysis was performed using Review Manager (RevMan) 5.4 software. For dichotomous outcomes, risk ratios (RR) with 95% confidence intervals (CI) were calculated. For continuous outcomes.Mean Difference (MD) was used for outcomes with identical units (e.g., NCV in m/s), while Standardized Mean Difference (SMD) was used for symptom scores due to varying scoring criteria. Heterogeneity between studies was assessed using the I² statistic: I² ≤ 50% was considered acceptable, and a fixed-effect model was applied; I² > 50% indicated substantial heterogeneity, and a random-effects model was used. For data with extremely high heterogeneity (e.g., NCV), a five-tiered progressive analytical strategy was employed (see **Figure 1**).

#### 2.5.1 Comprehensive processing of NCV data and the five-tiered analytical strategy

Given the extreme variability in nerves measured across studies (as visualized in **Table 1**), any attempt to calculate a ‘composite’ NCV value would be physiologically implausible and methodologically unsound, as it relies on the invalid assumption of independence between different nerve measurements. Therefore, we did not perform quantitative synthesis at this level. Instead, Tier 1 serves as a descriptive overview to demonstrate the extent of measurement heterogeneity, confirming the necessity of our tiered deconstructive approach. The specific calculation method and limitations are detailed in section 2.5.1 of the main text. Subsequently, the following five-tiered analysis was conducted:

**Table 1:** Heterogeneity in nerve conduction velocity measurement protocols across included studies.

Study (Year)	First author	Peroneal nerve		Median nerve		Tibial nerve		Sural nerve	Other nerves / Notes (e.g., Composite/Unspecified)
		MCV	SCV	MCV	SCV	MCV	SCV		
1 (2014)	Li Feng (35)	NR	NR	NR	NR	NR	NR	NR	Only "MNCV (m/s)" and "SNCV (m/s)" (composite/unspecified) reported.
2 (2016)	Lu Defu (16)	Reported	NR	NR	NR	Reported	NR	Reported	–
3 (2015)	Liu Zhanbing (17)	Reported	Reported	Reported	Reported	Reported	NR	NR	–
4 (2017)	Song Dan (18)	Reported	NR	Reported	Reported	Reported	NR	Reported	–
5 (2020)	Zu Lihua (19)	Reported	Reported	Reported	Reported	NR	NR	NR	–
6 (2019)	Lin Chenxin (20)	Reported	NR	NR	NR	NR	NR	Reported	–
7 (2016)	Yao Qi (21)	Reported	Reported	Reported	Reported	NR	NR	NR	–
8 (2020)	Jin Zhe (22)	NR	NR	NR	NR	NR	NR	NR	Only "MNCV(m/s)" and "SNCV (m/s)" (composite/unspecified) reported.
9 (2019)	Zhao Hua (23)	NR	NR	NR	NR	NR	NR	NR	Only "MNCV(m/s)" and "SNCV (m/s)" (composite/unspecified) reported.
10 (2015)	Guo Xingyang (24)	Reported	NR	Reported	Reported	Reported	NR	Reported	–
11 (2018)	Zhang Liyun (25)	Reported	Reported	Reported	Reported	NR	NR	NR	–
12 (2017)	Liu Aihua (26)	Reported	NR	Reported	Reported	Reported	NR	NR	Reported "SNCV" (unspecified).

MCV - Motor Conduction Velocity; SCV - Sensory Conduction Velocity; NR - Not Reported; MNCV - Motor Nerve Conduction Velocity (composite or unspecified); SNCV - Sensory Nerve Conduction Velocity (composite or unspecified).

Notes for this table:

1. Data Source: Information extracted from the provided Master Table and related nerve-specific worksheets (e.g., Common Peroneal Nerve MCV, Median Nerve SCV, Sural Nerve SCV). If MCV or SCV data for a specific nerve exists in the corresponding worksheet, it is marked as Reported.

2. Heterogeneity Demonstration: This table clearly demonstrates substantial measurement heterogeneity across studies. For example:

1. Only 8/12 studies reported Peroneal Nerve MCV.

2. Only 4/12 studies reported Peroneal Nerve SCV.

3. Only 3/12 studies reported Sural Nerve SCV.

4. 3 studies only reported composite "MNCV" and "SNCV" without specifying individual nerves.

Clinical Significance: This fundamental inconsistency in what was measured is key evidence in the Results section to justify why quantitative synthesis (meta-analysis) of an "overall" nerve conduction velocity effect is not methodologically sound. In the text, this table should be referenced: "As shown in Table X, the combination of nerves measured varied substantially across studies, with some reporting only composite scores. Therefore, pooling conduction velocity data from different nerves into a single 'NCV improvement' effect size is methodologically inappropriate."

Table generated based on data from "Data Extraction Table.xlsx". This table is intended for inclusion in systematic review manuscripts to demonstrate outcome measurement heterogeneity.

Tier 1: Preliminary Assessment Using Composite Indices.

First, pooled analysis of composite motor nerve conduction velocity (MCV) and sensory nerve conduction velocity (SCV) was performed to assess the overall effect and report heterogeneity levels. Predefined subgroup analyses based on treatment duration, Astragalus dose (<100g vs. ≥100g), and randomization method were also conducted to preliminarily explore sources of heterogeneity.

Tier 2: Focusing on Specific Nerve Measurements.

Based on the high heterogeneity identified in Tier 1, Tier 2 focused on independent pooled analyses of specific NCV indicators reported in ≥3 studies (common peroneal nerve MCV/SCV, median nerve MCV/SCV, sural nerve SCV). This aimed to identify variation introduced by measuring different nerves. The aforementioned subgroup analyses were repeated.

Tier 3: Sensitivity Testing and Robustness Assessment.

To assess the robustness of findings from Tier 2, Tier 3 performed “leave-one-out” sensitivity analyses on all pooled analyses from the first two tiers. This systematically examined the influence of individual studies on the overall effect estimate and heterogeneity, identifying the fragility of the evidence body and potential outliers.

Tier 4: In-depth Exploration of Core Intervention Variables.

Given that common peroneal nerve MCV showed relative robustness in Tier 3, Tier 4 focused on the core intervention variable—Astragalus dose. Using common peroneal nerve MCV as the outcome, subgroup analyses by Astragalus dose category (low: <30g, medium: 30-90g, high: ≥100g) and dose-effect meta-regression (linear and quadratic models) were performed to explore associations. It is critical to acknowledge that ‘dose’ here refers to the crude dry weight of the herb (grams), which is an imprecise surrogate for pharmacological exposure due to variability in bioactive compound concentrations.

Tier 5: Qualitative Synthesis and Pattern Recognition.

Due to fundamental challenges for quantitative pooling, Tier 5 abandoned quantitative synthesis and instead constructed a descriptive evidence matrix for qualitative synthesis of key studies. By integrating NCV results with intervention details and patient characteristics, this tier aimed to identify potential effective patterns and “pattern-syndrome correspondence” relationships, providing a basis for generating future research hypotheses.

Potential publication bias was assessed by visual inspection of funnel plots and Egger’s test (when the number of included studies was ≥10). A P-value < 0.05 was considered statistically significant for all analyses.

## 3 Results

### 3.1 Literature selection flow and characteristics of included studies

**Figure 2:**
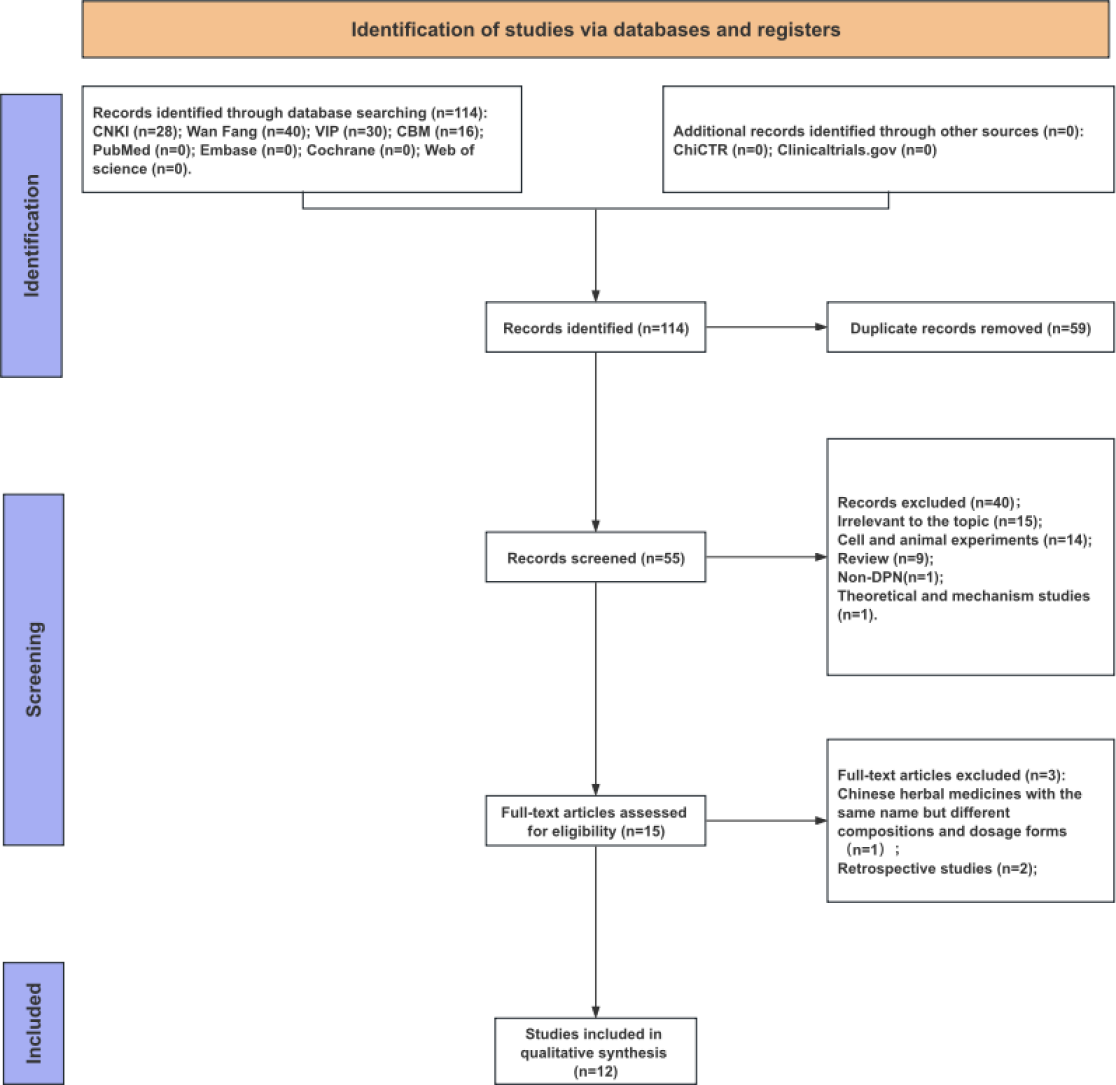
Flow diagram of studies selection process.

The initial search yielded 114 records. After deduplication and sequential screening, 12 RCTs (4–15) were finally included (literature selection flow is shown in **Figure 2** (PRISMA flowchart)). All studies were conducted in China and published between 2014 and 2020. The total sample size was 926 patients (468 in experimental groups, 458 in control groups). The mean age of patients ranged from 53 to 66 years. A treatment duration of 4 weeks was most common. The experimental groups received Buyang Huanwu Decoction (BYHWD) in addition to the control regimen, with Astragalus doses ranging from 10g to 120g. The baseline characteristics of the included studies (35–37) are detailed in **Table 2**.

**Table 2:** Baseline characteristics of included studies.

Study	Sample (T/C)	Gender (M/F)	Age	DM duration (Years)	DPN duration (Months)	Weeks	Intervention (T vs C)	Outcomes
Li et al., 2014 (15)	44/42	48/38	~66.0	6-15 (T), 6-12 (C)	NR	4	T: Basic therapy + ALA iv + BYHWD (Astragalus dose NR) C: Basic therapy + ALA iv	①
Lu et al., 2016 (16)	30/30	34/26	58.5	NR	~7.3 (T), ~7.2 (C)	2	T: Basic therapy + ALA iv + BYHWD (Astragalus 60g) C: Basic therapy + ALA iv	③, ④, ⑤, ⑥, ⑦
Liu et al., 2015 (17)	65/64	69/60	52.7	~6.4	~11.3	4	T: Basic therapy + ALA iv + Modified BYHWD (Astragalus 120g) C: Basic therapy + ALA iv	①, ②, ③, ④, ⑤, ⑥, ⑦
Song et al., 2017 (18)	30/30	34/26	61.9	NR	~117.6 (T), ~117.9 (C)	4 (2 + 2)	T: Basic therapy + ALA iv (2w) + Modified BYHWD (Astragalus 40g, 4w) C: Basic therapy + ALA iv (2w)	①, ③, ④, ⑤, ⑥, ⑦, ⑧, ⑨, ⑩, ⑬;
Zu et al., 2020 (19)	52/52	58/46	56.9	NR	~23.0 (T), ~23.4 (C)	8	T: Basic therapy + ALA po + BYHWD (Astragalus 120g) C: Basic therapy + ALA po	①, ④, ⑤, ⑥, ⑦, ⑧, ⑨, ⑩, ⑬;
Lin et al., 2019 (20)	57/57	52/62	57.6	~10.5	NR	4	T: Basic therapy + ALA iv + BYHWD (Astragalus 60g) C: Basic therapy + ALA iv	④, ⑤, ⑪, ⑫
Yao et al., 2016 (21)	40/40	41/39	57.8	NR	~93.6 (T), ~102.0 (C)	4	T: Basic therapy + ALA iv + BYHWD (Astragalus 100g) C: Basic therapy + ALA iv	①, ④, ⑤, ⑥, ⑦, ⑫
Jin et al., 2020 (22)	50/50	62/38	56.5	~7.0	NR	4.3 (30d)	T: Basic therapy + ALA iv + Modified BYHWD (Astragalus 30g) C: Basic therapy + ALA iv	①, ③, ④, ⑦, ⑫, ⑬;
Zhao et al., 2019 (23)	46/46	59/33	57.1	~12.3	~2.1	2	T: Basic therapy + ALA iv + Modified BYHWD (Astragalus 30g) C: Basic therapy + ALA iv	④, ⑦
Guo et al., 2015 (24)	30/30	31/29	51.3	~5.4	NR	2	T: Basic therapy + ALA iv + Modified BYHWD (Astragalus 40g) C: Basic therapy + ALA iv	①, ③, ④, ⑤, ⑥, ⑦
Zhang et al., 2018 (25)	62/62	69/55	58.4	~7.7	NR	3 (21d)	T: ALA iv + Modified BYHWD (Astragalus 10g + Scorpion, Centipede) C: ALA iv	①, ③, ④, ⑤, ⑥, ⑦, ⑭
Liu et al., 2017 (26)	42/42	40/44	58.3	~7.2	NR	4	T: Basic therapy + ALA iv + BYHWD (Astragalus 100g) C: Basic therapy + ALA iv	①, ④, ⑤, ⑥, ⑦

T: Trial group; C: Control group; DM: Diabetes mellitus; DPN: Diabetic peripheral neuropathy; NR: Not reported; Basic therapy: Included glycemic control, diet, exercise, and management of hypertension/hyperlipidemia as needed; ALA: α-Lipoic acid; iv: Intravenous infusion; po: Oral administration; BYHWD: Buyang Huanwu Decoction (补阳还五汤).

Outcome indices:① Overall effective rate;② TCSS score;③ TCM syndrome score (total); ④ Motor nerve conduction velocity (MCV);⑤ Sensory nerve conduction velocity (SCV);⑥ Peroneal nerve MCV;⑦ Peroneal nerve SCV;⑧ Median nerve MCV;⑨ Median nerve SCV;⑩ Sural nerve SCV;⑪ Vibration perception threshold (VPT);⑫ Glycated hemoglobin (HbA1c);⑬; Oxidative stress markers (SOD, MDA, T-AOC);⑭ Urinary N-acetyl-β-D-glucosaminidase (NAG)

Age and duration data are presented as mean or approximate mean based on available data from the extraction sheet. The "~" symbol indicates an estimated value derived from the reported data (e.g., mean calculated from a range).

DPN Duration Unit Correction: All DPN duration data without explicit units in the original extraction sheet have been interpreted and presented as months, per the user's instruction. Data explicitly reported in years have been converted to months (×12).

### 3.2 Risk of bias assessment

**Figure 3:**
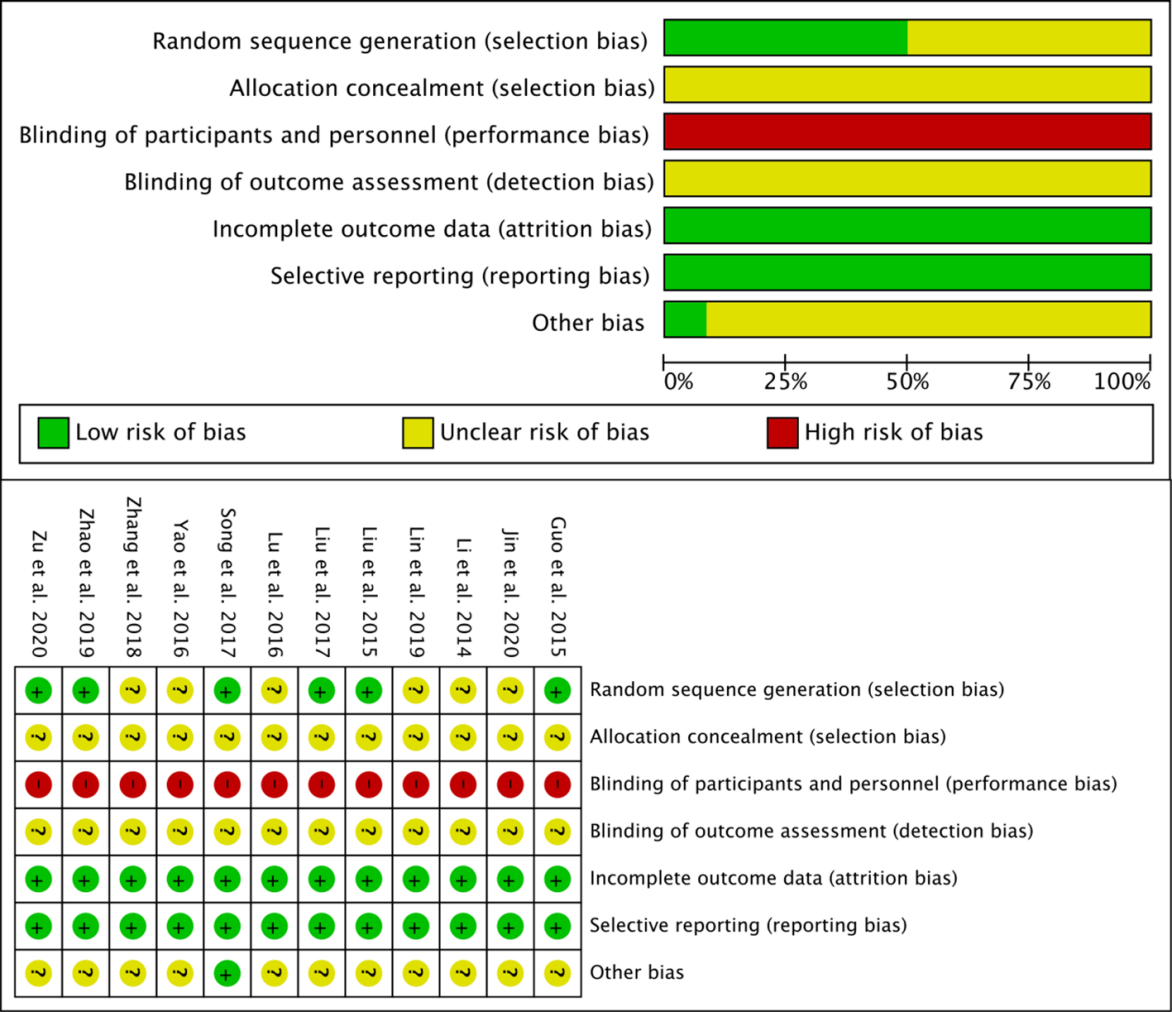
Summary of the risk of bias assessment for included studies.

The overall methodological quality of the included studies was moderate. All studies mentioned randomization; seven described specific randomization methods (low risk), while five only mentioned “randomized” (unclear risk). None of the studies clearly described allocation concealment (unclear risk). Only one study mentioned blinding of outcome assessors. All studies reported complete outcome data (low risk). A summary of the risk of bias assessment is presented in **Figure 3**.

### 3.3 Meta-analysis results

#### 3.3.1 Overall response rate and TCM syndrome scores

**Figure 4:**
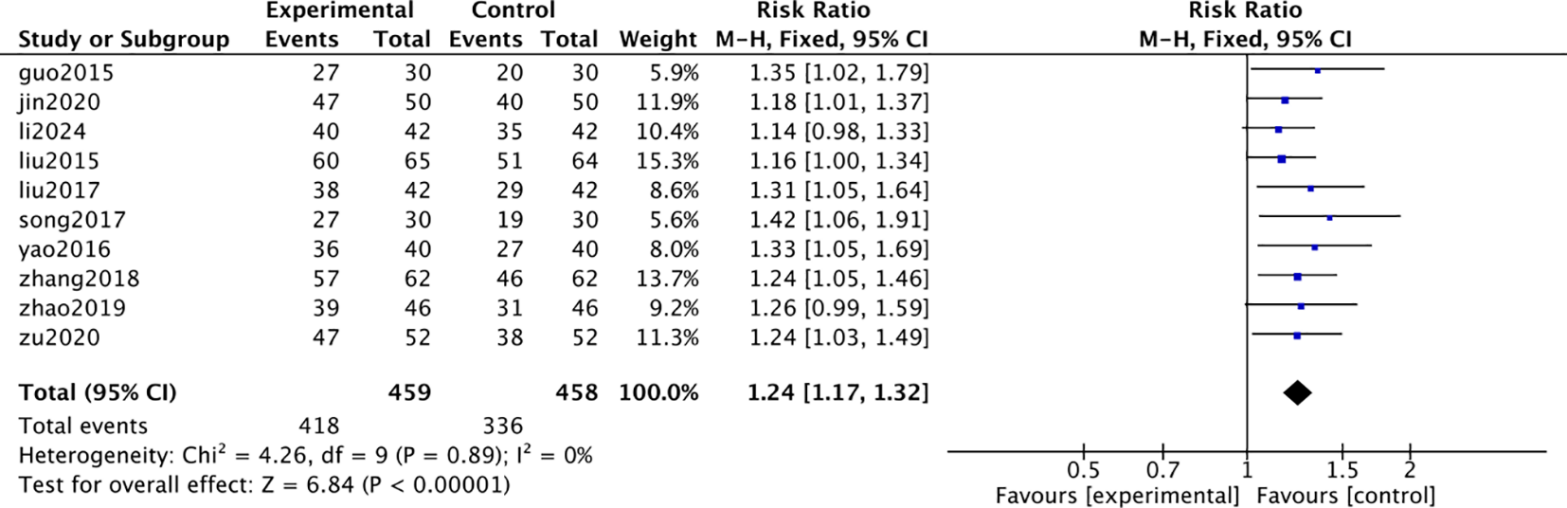
Forest plot of the meta-analysis for overall response rate.

To address inconsistency in efficacy evaluation criteria (e.g., cured, markedly effective, effective, ineffective) across studies, efficacy endpoints were unified into a dichotomous variable for pooled analysis. Specifically, cases reported as “cured,” “markedly effective,” or “effective” were categorized as “effective,” and “ineffective” cases as “ineffective.” Ten studies reported the overall response rate. The combined therapy group showed a significantly higher overall response rate than the control group (RR = 1.24, 95% CI: 1.17-1.32, P < 0.00001), with no heterogeneity (I² = 0%). **Figure 4** (Forest plot for overall response rate) shows a consistent direction of effect. Four studies reported TCM syndrome total scores, indicating superior improvement in the combined therapy group (SMD = -0.76, 95% CI: -0.98 to -0.54, P < 0.001). Analyses of individual symptoms like limb numbness and shortness of breath also favored the combined therapy.

#### 3.3.2 Systematic evaluation of nerve conduction velocity: results of the five-tiered progressive analysis

Confronted with extremely high heterogeneity for NCV indicators (I² > 90%), this study did not confine itself to reporting a potentially misleading pooled effect size. Instead, it employed the pre-defined five-tiered progressive analytical framework (**Figure 1**). This framework aimed to deconstruct the sources of heterogeneity layer by layer: from an overall assessment using composite indices (Tier 1), to focusing on specific nerves to identify measurement bottlenecks (Tier 2), testing result robustness via sensitivity analysis (Tier 3), then delving into the complex effects of the core intervention variable (Astragalus dose) (Tier 4), and finally switching to descriptive evidence synthesis when quantitative pooling was unfeasible (Tier 5). This process not only assessed efficacy but also aimed to reveal the fragility and sources of uncertainty within the current evidence body.

Tier 1: Composite NCV Analysis – Revealing Fundamental, Unexplained Heterogeneity.

**Figure 5:**
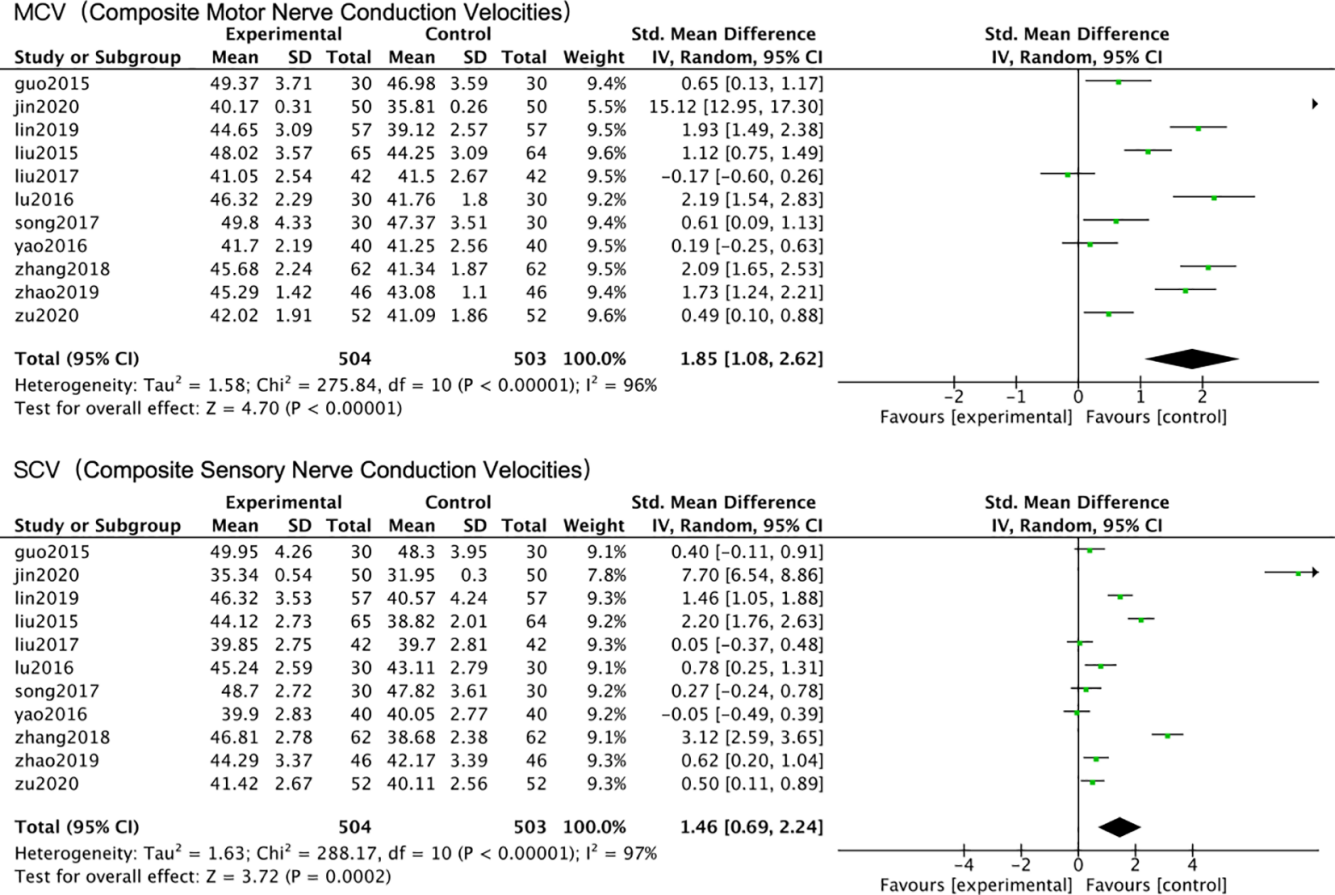
Forest plot of the meta-analysis on composite motor and sensory nerve conduction velocities.

To preliminarily integrate data from different nerves measured across studies, composite motor (Composite MCV) and sensory (Composite SCV) nerve conduction velocities were calculated for each study for pooled analysis. For each study that reported nerve conduction velocities of multiple nerves of the same type (e.g., motor nerves or sensory nerves), we calculated the simple arithmetic mean of the measured values as the composite nerve conduction velocity and estimated the composite standard deviation using the formula of the square root of the sum of squares of the standard deviations of each nerve divided by the number of nerves, which was then used for subsequent meta−analysis. As pre-specified, quantitative synthesis was not attempted due to the invalidating heterogeneity in measurement protocols. A descriptive summary (**Table 1**) shows that studies measured widely different sets of nerves (e.g., only 4/12 reported sural SCV). Any notional pooled estimate derived from such incommensurable data would be statistically unreliable, as evidenced by an I² value >96% in exploratory calculations (**Figure 5**). Due to this substantial heterogeneity, a random-effects model was employed for all pooled analyses in this tier. This confirms the necessity of proceeding to nerve-specific analyses. This suggests great uncertainty in the pooled effect sizes. Subsequent pre-defined subgroup analyses based on treatment duration, Astragalus dose (<100g vs. ≥100g), and randomization method (**Table 3**) all failed to substantially reduce heterogeneity (all subgroup I² > 90%), indicating that these conventional factors cannot explain the fundamental differences present in the evidence body. “The composite NCV analysis revealed fundamental, unexplained heterogeneity, prompting us to proceed to Tier 2 analysis.” .

**Table 3:** Results of exploratory subgroup analyses for composite sensory and motor nerve conduction velocities.

Composite MCV					
Subgrouping dimension	Subgroup category	No. of comparisons	Pooled SMD (95% CI)	I² within subgroup	P-value for subgroup difference
Treatment duration	≤ 30day	10	2.04 [1.18, 2.90]	97%	0.001
	> 30day	1	0.49 [0.10, 0.88]	—	
Astragalus dose	< 100g	7	2.90 [1.73, 4.07]	86%	0.0002
	≥ 100g	4	0.41 [-0.14, 0.96]	97%	
Randomization method	Low risk	5	0.75 [0.15, 1.36]	90%	0.003
	High/unclear risk	6	3.16 [1.70, 4.62]	98%	
Composite SCV					
Subgrouping dimension	Subgroup category	No. of comparisons	Pooled SMD (95% CI)	I² within subgroup	P-value for subgroup difference
Treatment duration	≤ 30day	10	1.57 [0.71, 2.44]	97%	0.03
	> 30day	1	0.50 [0.11, 0.89]	—	
Astragalus dose	< 100g	7	1.96 [0.82, 3.11]	97%	0.09
	≥ 100g	4	0.67 [-0.32, 1.66]	95%	
Randomization method	Low risk	5	0.73 [-0.00, 1.46]	93%	0.08
	High/unclear risk	6	2.15 [0.74, 3.57]	98%	

Tier 2: Specific Nerve NCV Analysis – Highlighting the Bottleneck of Missing Standardization.

**Figure 6:**
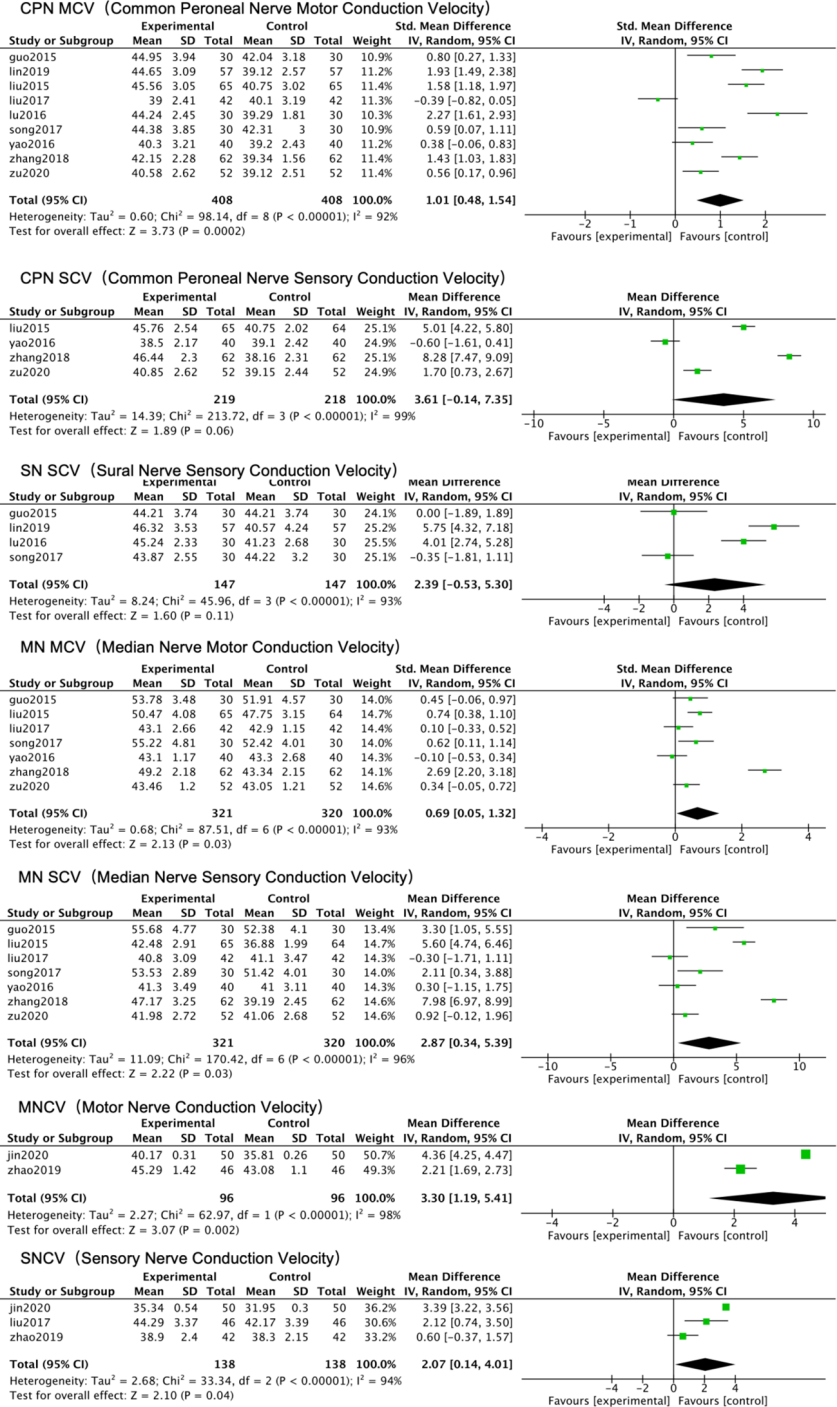
Forest plot of the meta-analysis for nerve conduction velocity (NCV) of specific nerves.

Given the excessive heterogeneity of composite indices, we turned to analyzing specific NCV measures reported in ≥3 studies. Results showed that only the pooled effect size for common peroneal nerve MCV exhibited relatively stable significance (SMD = 1.01, 95% CI: 0.48-1.54, P < 0.01), although I² remained high at 92% (**Figure 6**). A random-effects model was used for this analysis. For other nerve-specific indicators, such as median nerve MCV/SCV, common peroneal nerve SCV, and sural nerve SCV, the confidence intervals of the pooled results were extremely wide, and heterogeneity (I² range 93%-99%) was too high to allow any reliable interpretation of the effect (**Figure 6**). Due to the excessive heterogeneity, a random-effects model was employed for all these analyses. Subgroup analyses repeated for all specific nerve indicators similarly failed (**Table 4**). The core finding of this tier is that the lack of uniformity across studies in the nerves measured (e.g., common peroneal vs. median nerve), measurement sites, and methods is the direct technical cause preventing data synthesis and generating unexplainable heterogeneity. The fact that common peroneal nerve MCV became a relatively analyzable indicator precisely because its reporting was relatively concentrated, partially reducing the noise introduced by measurement variation. “The specific nerve analysis highlighted the bottleneck of missing measurement standardization, while also identifying common peroneal nerve MCV as a relatively analyzable indicator, laying the foundation for in-depth exploration of intervention effects.”

**Table 4:** Table of subgroup analysis for the meta-analysis of nerve-specific conduction velocity (NCV).

Common peroneal nerve MCV					
Subgrouping dimension	Subgroup category	No. of comparisons	Pooled SMD (95% CI)	I² within subgroup	P-value for subgroup difference
Treatment Duration	≤ 30day	8	2.91 [1.39, 4.42]	93%	0.12
	> 30day	1	1.46 [0.47, 2.45]	—	
Astragalus Dose	< 100g	5	3.73 [2.38, 5.09]	85%	0.13
	≥ 100g	4	1.58 [-0.82, 3.98]	93%	
Randomization Method	Low risk	4	1.82 [-0.72, 4.35]	94%	0.27
	High/unclear risk	5	3.94 [1.95, 5.02]	80%	
Common peroneal nerve SCV					
Treatment Duration	≤ 30day	3	4.24 [-0.51, 8.99]	99%	0.30
	> 30day	1	1.70 [0.73, 2.67]	—	
Astragalus Dose	< 100g	1	8.28 [7.47, 9.09]	97%	0.0003
	≥ 100g	3	2.05 [-1.26, 5.36]	99%	
Randomization Method	Low risk	2	3.37 [0.12, 6.61]	93%	0.92
	High/unclear risk	2	3.85 [-4.86, 12.55]	99%	
Sural nerve SCV					
Treatment Duration	≤ 30day	4	2.39 [-0.53, 5.30]	93%	—
	> 30day	0	—	—	
Astragalus Dose	< 100g	3	3.32 [0.38, 6.26]	91%	0.03
	≥ 100g	1	-0.35 [-1.81, 1.11]	—	
Randomization Method	Low risk	1	-0.35 [-1.81, 1.11]	—	0.03
	High/unclear risk	3	3.32 [0.38, 6.26]	91%	
Median nerve MCV					
Treatment Duration	≤ 30day	6	2.20 [-0.18, 4.59]	96%	0.15
	> 30day	1	0.41 [-0.05, 0.87]	—	
Astragalus Dose	< 100g	3	3.64 [0.82, 6.47]	88%	0.05
	≥ 100g	4	0.67 [-0.24, 1.59]	80%	
Randomization Method	Low risk	3	2.53 [-1.91, 6.96]	98%	0.59
	High/unclear risk	4	1.27 [0.11, 2.43]	81%	
Median nerve SCV					
Treatment Duration	≤ 30day	6	3.20 [0.40, 6.00]	96%	0.14
	> 30day	1	0.92 [-0.12, 1.96]	—	
Astragalus Dose	< 100g	3	4.53 [0.39, 8.66]	95%	0.22
	≥ 100g	4	15.07 [-1.29, 31.43]	100%	
Randomization Method	Low risk	4	2.11 [-0.83, 5.04]	100%	0.56
	High/unclear risk	3	3.88 [-1.39, 9.16]	97%	
Sensory NCV					
Treatment Duration	≤ 30day	3	2.63 [-0.44, 5.70]	97%	—
	> 30day	0	—	—	
Astragalus Dose	< 100g	2	1.28 [-0.20,5.76]	68%	0.09
	≥ 100g	1	5.10 [4.44, 5.76]	—	
Randomization Method	Low risk	2	2.87 [-1.54, 7.27]	98%	0.75
	High/unclear risk	1	2.12 [0.74, 3.50]	—	

Tier 3: Sensitivity Analysis – Confirming the General Fragility of the Evidence Body and the Relative Robustness of Common Peroneal Nerve MCV.

To assess the robustness of pooled results, “leave-one-out” sensitivity analyses were performed for all analyses in Tiers 1 and 2. Overall, the pooled effect sizes and heterogeneity for most NCV indicators were sensitive to the omission of individual studies, confirming the general fragility of the evidence body. For example, in the median nerve MCV analysis, omitting (13) reduced I² from 97% to 76% and reversed the conclusion from “no statistical significance” to “significant improvement” (MD = 1.01, 95% CI: 0.14-1.88, P = 0.02), indicating this study had a critical impact on the result.

**Figure 7:**
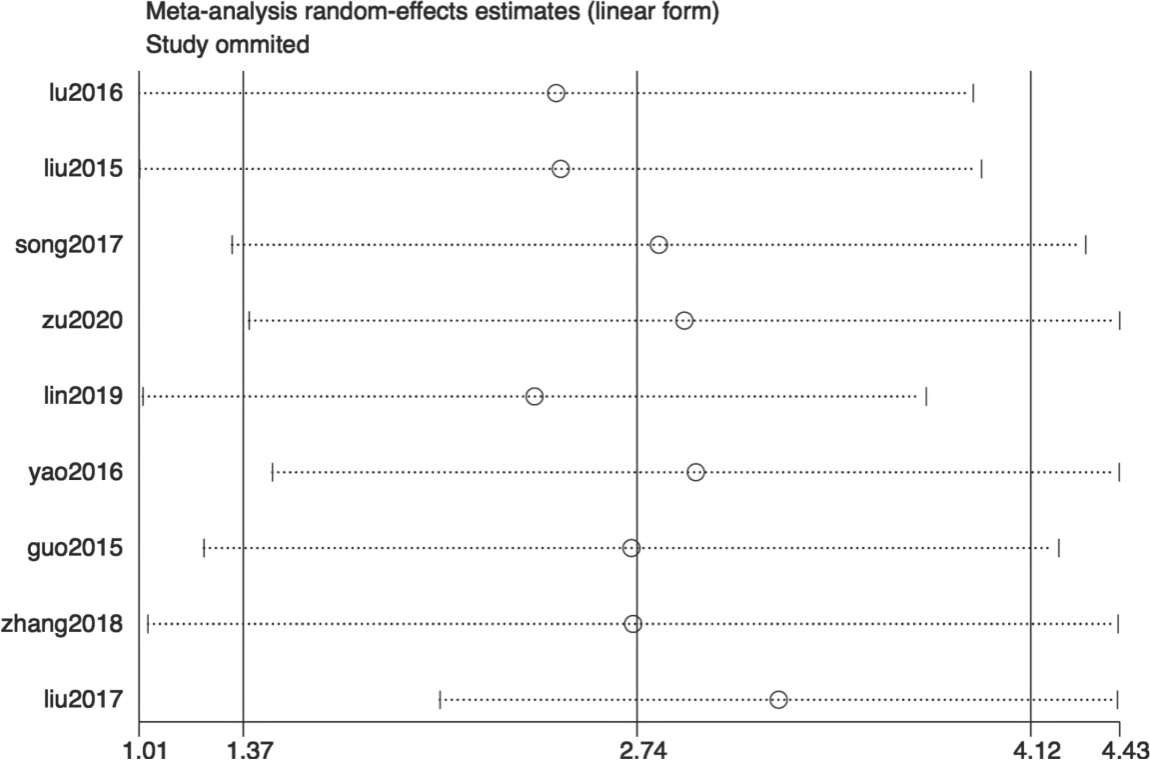
Sensitivity analysis of the common peroneal nerve motor conduction velocity (MCV): changes in effect size upon sequential study omission.

In contrast, the direction of effect (beneficial trend) for common peroneal nerve MCV was robust to sensitivity analysis. However, the point estimate of the effect size (SMD ≈ 1.01) remained unstable across sensitivity iterations (range: 0.86 – 1.18), and critically, the heterogeneity persisted at very high levels (I² 87%-93%). Consistent with the approach in previous tiers, a random-effects model was maintained for these analyses. This indicates that while the trend is consistent, the magnitude of the effect cannot be estimated with precision from the current evidence. This reinforces the conclusion from the first two tiers that “most NCV quantitative evidence is fragile and highly heterogeneous,” while also providing a basis for exploring sources of heterogeneity (e.g., intervention variables) in subsequent analyses. The leave-one-out sensitivity analysis (**Supplementary Material S6**, **Figure 7**) demonstrated that the pooled effect size for common peroneal nerve MCV remained statistically significant (**Table 5**) regardless of individual study exclusion, confirming robustness. “Sensitivity analysis confirmed the general fragility of the evidence body, while also verifying the relative robustness of the common peroneal nerve MCV result, providing a basis for subsequent analysis focusing on intervention variables.” .

**Table 5:** Results of the leave-one-out sensitivity analysis for the improvement effect (SMD) of common peroneal nerve motor conduction velocity (MCV).

Study omitted	No. of studies	Pooled SMD (95% CI)	I²	P value
Guo 2015 (24)	8	1.03 [0.44, 1.62]	93%	0.0006
Lin 2019 (20)	8	0.89 [0.35, 1.43]	91%	0.001
Liu 2015 (17)	8	0.94 [0.36, 1.51]	92%	0.002
Liu 2017 (26)	8	1.18 [0.73, 1.64]	87%	<0.00001
Lu 2016 (16)	8	0.86 [0.33, 1.39]	91%	0.001
Song 2017 (18)	8	1.06 [0.47, 1.65]	93%	0.0004
Yao 2016 (21)	8	1.09 [0.51, 1.67]	92%	0.0002
Zhang 2018 (25)	8	0.96 [0.36, 1.55]	92%	0.002
Zu 2020 (19)	8	1.07 [0.47, 1.67]	93%	0.0005

SMD, standardized mean difference; CI, confidence interval.

The consistency in the direction of effect and statistical significance across all analyses supports the robustness of the common peroneal nerve MCV as an outcome. The persistently high I² values indicate that heterogeneity is an inherent characteristic of the study population

Tier 4: Analysis of the Complex Effect of Astragalus Dose – Discoveries Beyond a Linear Relationship.

**Figure 8:**
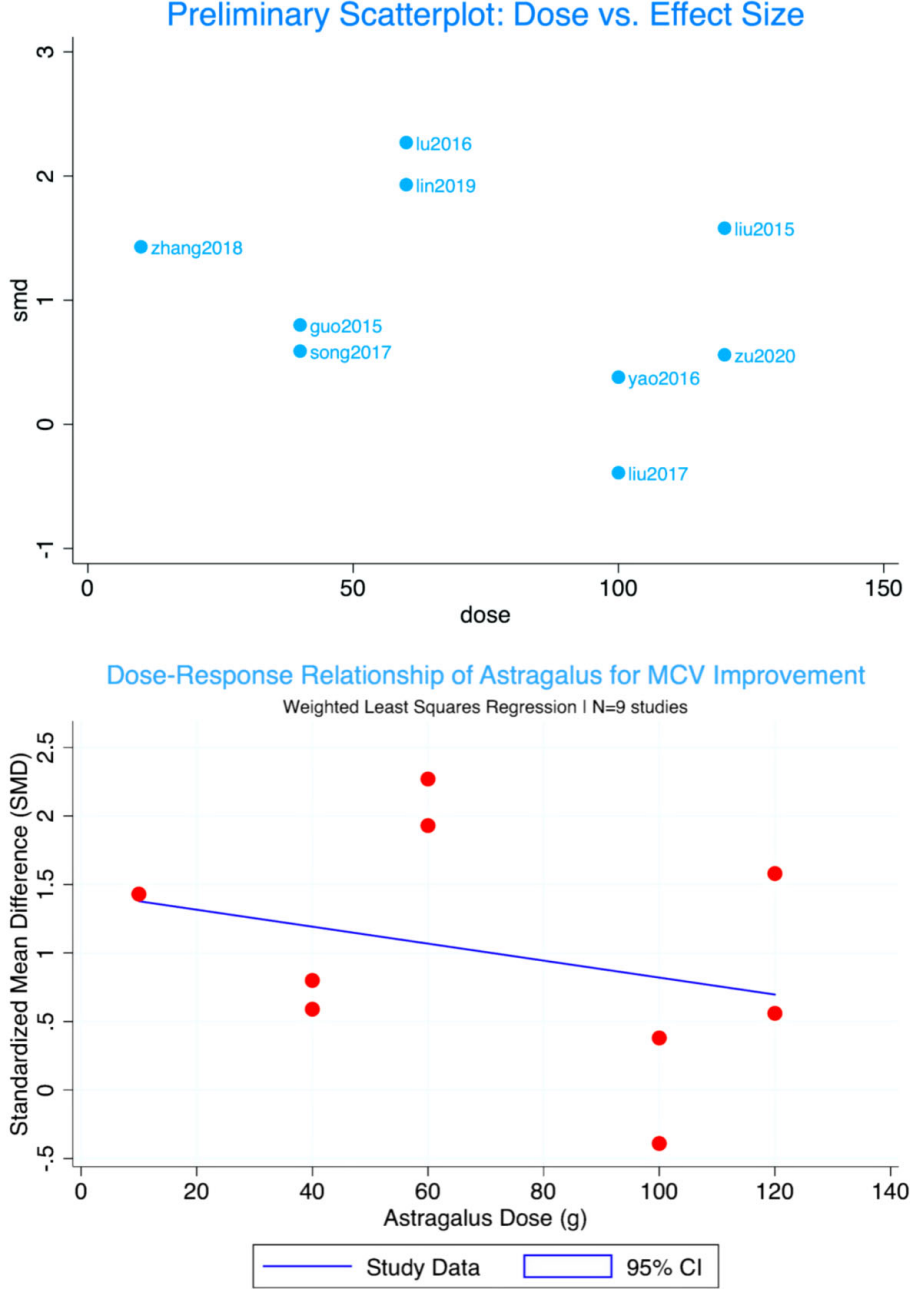
Dose-effect relationship between astragalus dose and improvement in common peroneal nerve MCV (standardized mean difference, SMD): scatter plot with non-linear regression curve.

Based on the Tier 3 results, we focused on the relatively robust common peroneal nerve MCV to explore the influence of the core intervention variable—Astragalus dose. Subgroup analysis by dose category (low: <30g, medium: 30-90g, high: ≥100g) showed the largest effect size in the medium-dose group (SMD = 1.39), while the high-dose group showed non-significant effects and extremely high within-group heterogeneity (I² = 93.3%). A random-effects model was applied for this subgroup analysis due to the substantial heterogeneity. However, dose-effect meta-regression (linear and quadratic models) showed no statistical significance (P > 0.40), with negative model explanatory power. “The dose-effect meta-regression showed no statistical significance (P > 0.40), indicating that traditional linear models cannot sufficiently explain the relationship between Astragalus dose and efficacy.” As shown in **Figure 8**, data points were widely scattered, suggesting the potential existence of unmeasured key moderating variables. Integrating TCM theory, we propose the ‘pattern-syndrome correspondence’ hypothesis: the efficacy of high-dose Astragalus may highly depend on its application to matched ‘severe qi deficiency and marked blood stasis’ patterns, and whether the 配伍 (compatibility) of blood-activating and collateral-unblocking herbs in the prescription is appropriate. “The analysis focusing on Astragalus dose did not reveal a simple linear relationship but suggested more complex effect patterns, guiding us toward qualitative integration in Tier 5.” .

Tier 5: Descriptive Evidence Synthesis – From Quantitative Pooling to Qualitative Pattern Recognition.

**Figure 9:**
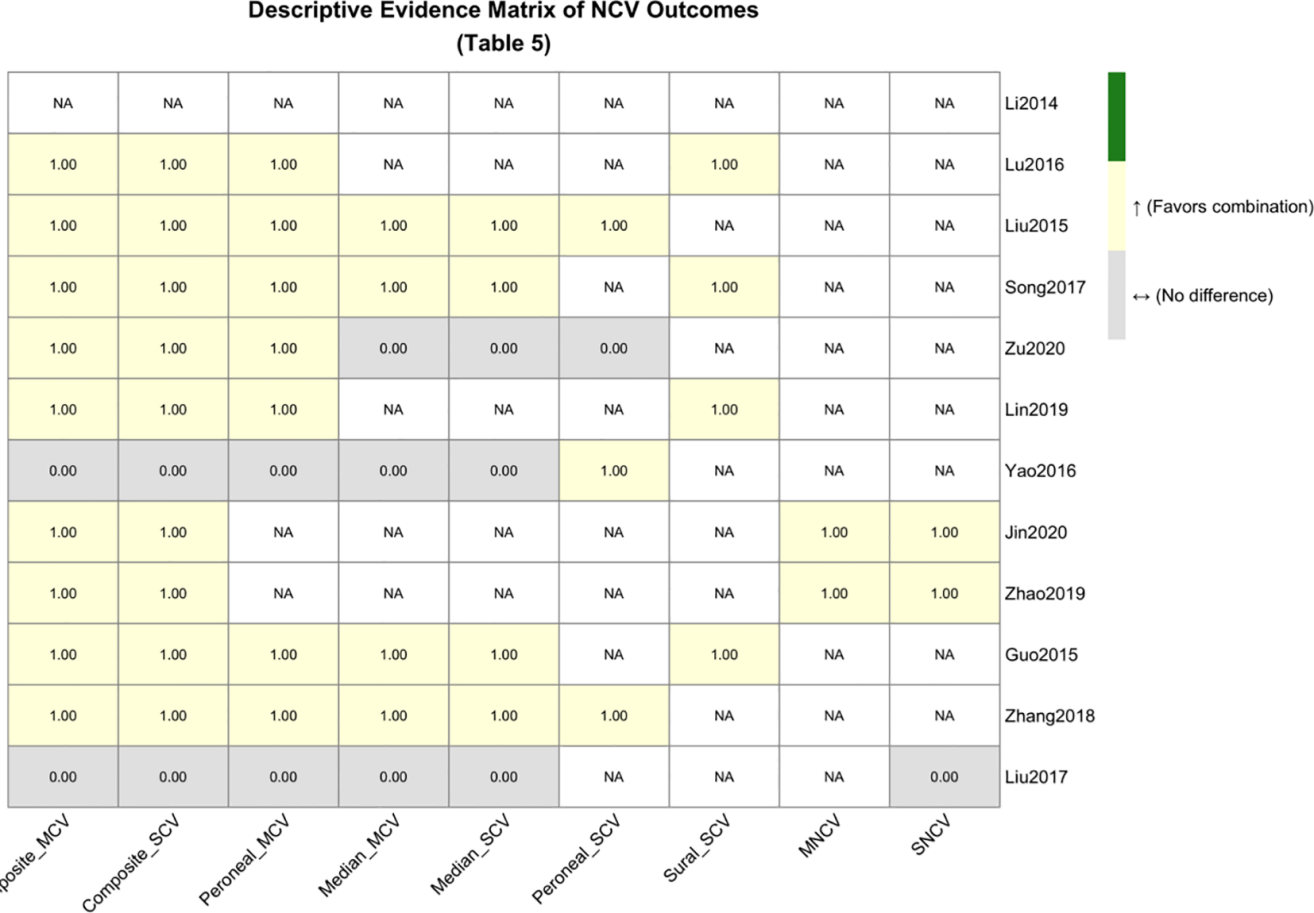
Heatmap of the descriptive evidence matrix for nerve conduction velocity outcomes.

Given that the aforementioned analyses indicated unreliable conclusions from quantitative pooling, we refrained from providing a single effect value and instead constructed a descriptive evidence matrix (**Table 6**) for qualitative synthesis of key studies. This matrix integrated information from each study on nerve conduction velocity, Astragalus dose, patient baseline characteristics (e.g., disease duration), and formula compatibility. Several typical patterns were summarized: e.g., “classic dose (60g) effective” (10), “high dose effective when matching long-duration blood stasis pattern” (9), “high dose ineffective when pattern-syndrome mismatched” (8), and “dissociation between symptom improvement and electrophysiological improvement” (12). This qualitative synthesis suggests that “BYHWD combined with ALA” in clinical research is actually a highly heterogeneous composite intervention, and its efficacy is the result of the complex interaction of multiple factors such as dose, pattern, and compatibility. The descriptive evidence matrix synthesizing NCV outcomes is presented in **Table 6**, and the corresponding visual heatmap is shown in **Figure 9**.

**Table 6:** Descriptive evidence matrix of nerve conduction velocity outcomes across included studies.

Study (Year)	Composite MCV	Composite SCV	Peroneal MCV	Median MCV	Median SCV	Peroneal SCV	Sural SCV	MNCV^†^	SNCV^†^
Li, 2014 (15)	-	-	-	-	-	-	-	-	-
Lu, 2016 (16)	↑	↑	↑*	-	-	-	↑	-	-
Liu, 2015 (17)	↑	↑	↑*	↑	↑	↑	-	-	-
Song, 2017 (18)	↑	↑	↑*	↑	↑	-	↑	-	-
Zu, 2020 (19)	↑	↑	↑*	↔	↔	↔	-	-	-
Lin, 2019 (20)	↑	↑	↑*	-	-	-	↑	-	-
Yao, 2016 (21)	↔	↔	↔*	↔	↔	↑	-	-	-
Jin, 2020 (22)	↑	↑	-	-	-	-	-	↑	↑
Zhao, 2019 (23)	↑	↑	-	-	-	-	-	↑	↑
Guo, 2015 (24)	↑	↑	↑*	↑	↑	-	↑	-	-
Zhang, 2018 (25)	↑	↑	↑*	↑	↑	↑	-	-	-
Liu, 2017 (26)	↔	↔	↔*	↔	↔	-	-	-	↔

↑: Favors combination therapy (experimental group significantly better than control, P < 0.05, based on study-reported between-group comparison or meta-analysis effect estimate with 95% CI not crossing zero).

↔: No statistically significant difference between groups (P ≥ 0.05).

↓: Favors control group (significantly better than combination therapy). Note: Not observed in the current dataset.

-: Not reported or insufficient data for comparison.

† MNCV: Motor Nerve Conduction Velocity; SNCV: Sensory Nerve Conduction Velocity. These refer to composite or unspecified nerve measurements as reported in the original studies.

* Peroneal MCV results are derived from the meta-analysis of 9 studies with complete data, as detailed in Section 3.3.2.

#### 3.3.3 Other secondary outcomes and safety

**Figure 10:**
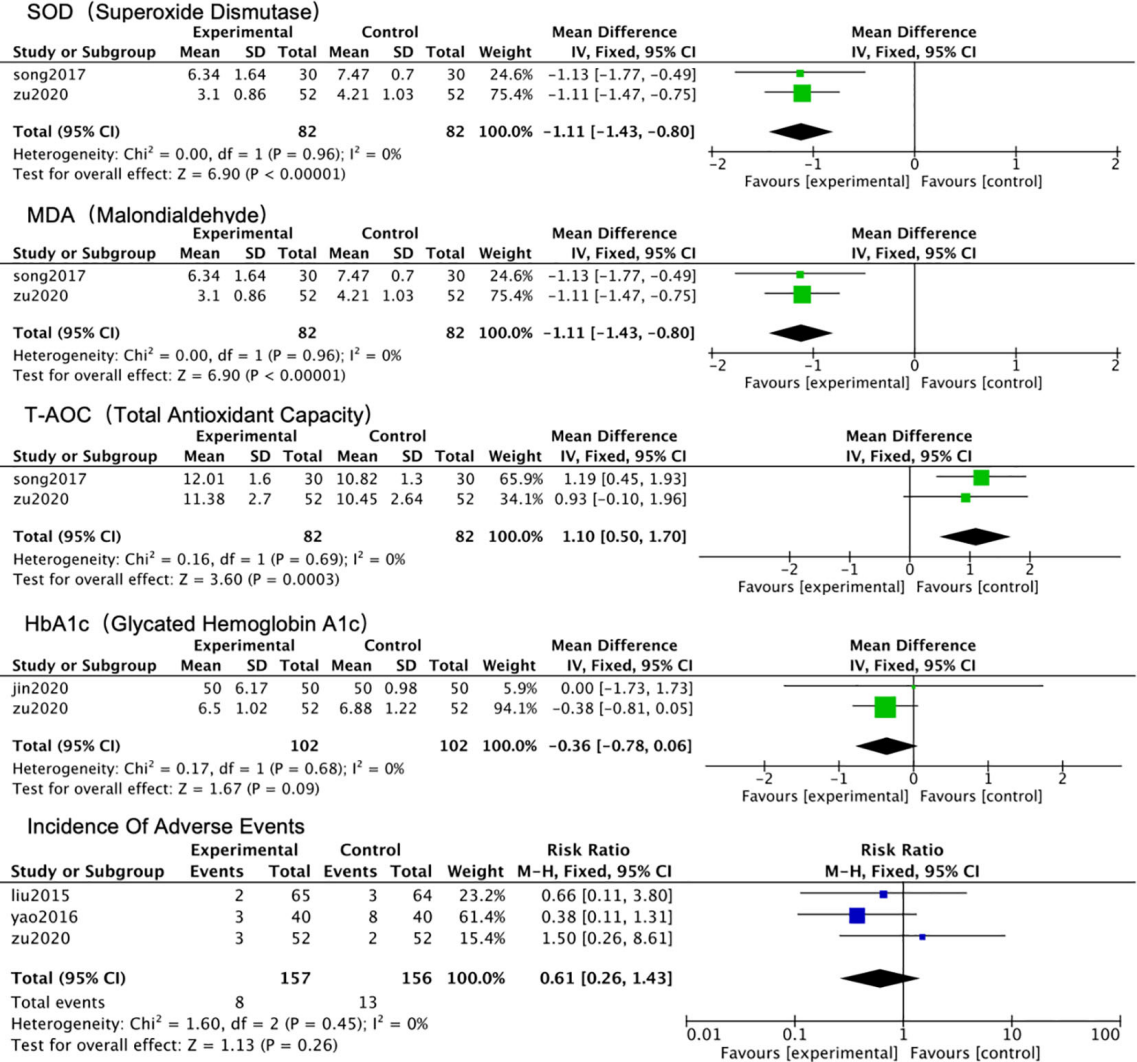
Forest plot of the meta-analysis for other secondary outcomes and safety.

As illustrated in **Figure 9** Forest Plot of the Meta-analysis for Other Secondary Outcomes and Safety, descriptive analysis of oxidative stress markers (SOD, MDA, T-AOC) reported in 2 studies suggested a potential advantage for the combined therapy. Two studies reporting the glycemic control indicator HbA1c showed no additional benefit from the combined therapy. Regarding safety, 3 studies reported adverse events. Adverse events were reported in 3 studies. Specific symptoms were mild and transient, including nausea and dry mouth (12), dizziness/head distension (9), and mild skin rash (15). No serious adverse events were observed. (**Figure 10**).

### 3.4 Publication bias

**Figure 11:**
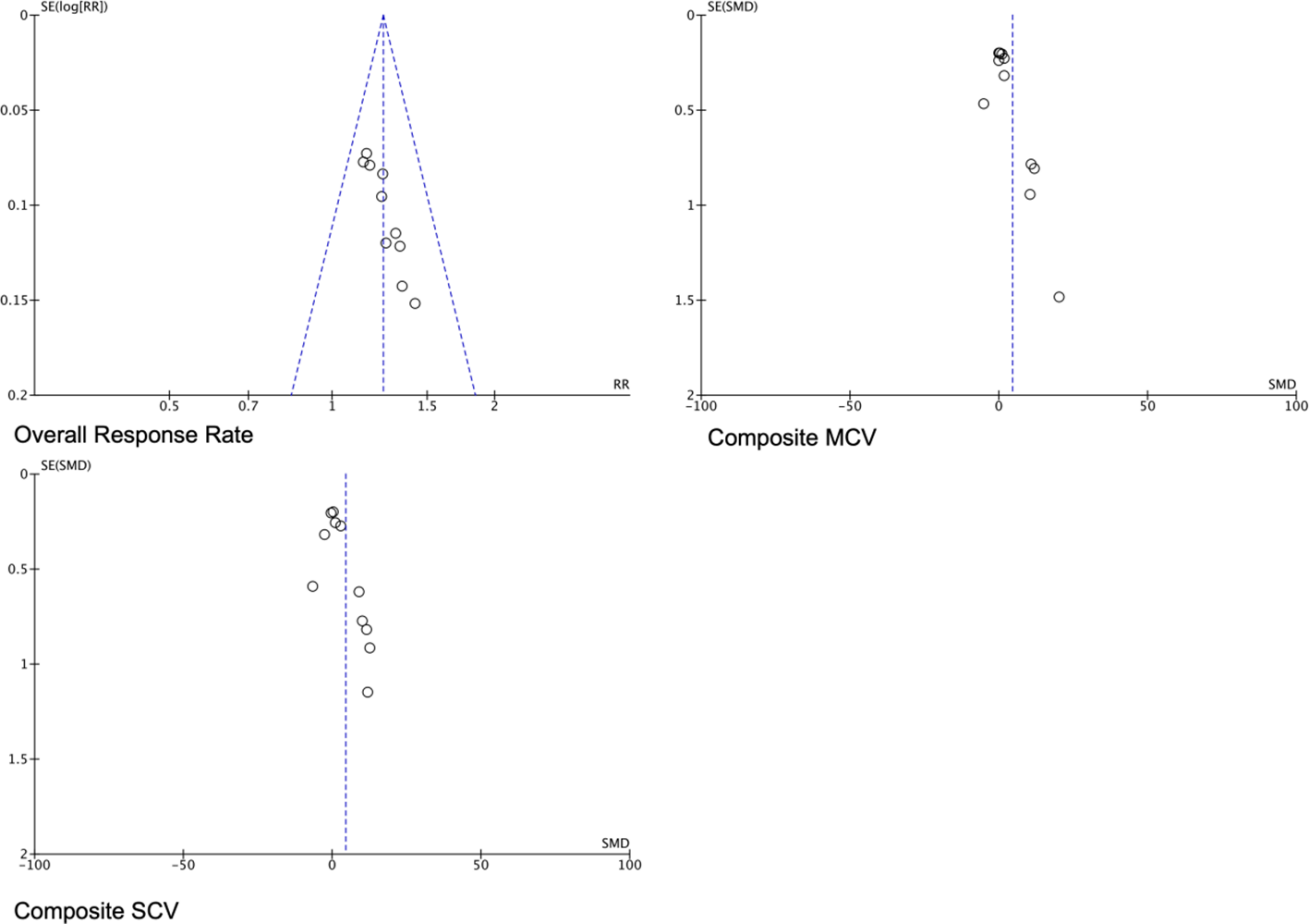
Funnel plot for assessing publication bias.

Publication bias was assessed for the primary outcome (overall response rate), which was reported in 10 studies. Visual inspection of the funnel plot (**Figure 11**) revealed no significant asymmetry, suggesting the absence of substantial publication bias. Egger’s regression test further supported this finding. These results indicate that the pooled effect estimate for overall response rate is unlikely to be biased by the selective publication of small studies with positive results.

## 4 Discussion

### 4.1 Principal findings: a tale of two evidence streams

This meta-analysis provides clear evidence that Buyang Huanwu Decoction (BYHWD) combined with α-lipoic acid (ALA) significantly improves the overall response rate and effectively alleviates Traditional Chinese Medicine (TCM) clinical symptoms, particularly the core patterns of qi deficiency and blood stasis, in patients with DPN, without increasing safety risks. This offers an effective and safe integrated Chinese and Western medicine option for clinically relieving subjective suffering and enhancing quality of life. However, regarding the key objective indicator of neural structural repair—nerve conduction velocity (NCV)—a more complex picture was revealed through systematic deconstruction using our innovative five-tiered progressive analytical framework. The analysis indicates that although positive trends were observed under specific conditions (e.g., for the relatively consistently reported common peroneal nerve MCV), the fundamental, systematic heterogeneity within the evidence body (I² > 96%) renders any simple quantitative conclusion of “effective” or “ineffective” arbitrary and unreliable (3, 33). The core value of this study lies in not avoiding or simplistically ignoring this heterogeneity. Instead, we transformed it from a purely methodological obstacle into an opportunity to deepen the understanding of the combination therapy’s characteristics and to reflect on the deficiencies of current research through a systematic analytical process.

### 4.2 A methodological autopsy: deconstructing the sources of heterogeneity

This study systematically deconstructed the heterogeneity within the efficacy evidence for BYHWD combined with ALA in treating DPN through the pre-defined five-tiered progressive analytical framework. This process not only revealed key methodological bottlenecks in current research but also provided a new perspective for understanding the complexity of integrated Chinese and Western medicine treatment.

#### 4.2.1 Tier 1 & 2: the primary bottleneck – non-standardized measurement

The first and second tiers of analysis jointly exposed the fundamental defects in the measurement of objective outcome indicators in current studies. The extremely high heterogeneity (I² > 96%) presented in the composite NCV analysis, coupled with the failure of conventional subgroup analyses (e.g., by treatment duration, dose grouping), suggests that the source of heterogeneity is not merely simple clinical variable differences (3, 33). Notably, despite the use of a random-effects model to account for this variability, the heterogeneity remained persistently high, indicating that the inconsistency stems from more fundamental methodological differences rather than random variation. The second-tier analysis focusing on specific nerves further concretized the problem: aside from the relatively consistently reported common peroneal nerve MCV, the pooled results for other indicators like median nerve and sural nerve NCV were uninterpretable due to excessively high heterogeneity (I² >93%). This directly indicates that the lack of uniformity across studies in the choice of measured nerves, measurement sites, equipment, and operational procedures is the primary technical cause preventing data synthesis and generating unexplainable variation. The fact that common peroneal nerve MCV became a relatively analyzable indicator precisely proves the importance of standardized measurement—its reporting was relatively concentrated, objectively reducing measurement noise (1, 32). This finding provides a mandatory methodological recommendation for all future DPN efficacy research: a standardized measurement protocol for core electrophysiological indicators (including at least 1-2 standardized nerves, such as the common peroneal nerve) must be pre-registered and reported in detail within the study protocol.

#### 4.2.2 Tier 3: the fragility of the evidence base

The third-tier sensitivity analysis provided critical insight for assessing evidence quality. The analysis showed that the pooled effect sizes for most NCV indicators were highly sensitive to the omission of individual studies, confirming the general fragility of the current evidence body. Under the random-effects model framework, this sensitivity suggests that the evidence base is dominated by a few influential studies, underscoring the need for larger, more robust trials. For instance, the conclusion for median nerve MCV could reverse upon the omission of a particular study. In stark contrast, the improvement effect (SMD) for common peroneal nerve MCV maintained robust direction and statistical significance in the sensitivity analysis (all 95% CIs excluded 0 after any omission). However, its heterogeneity (I²) remained persistently high (87%-93%) after omitting any study. This paradoxical phenomenon of “robust effect yet strong heterogeneity” is significant: it suggests that common peroneal nerve MCV may indeed be an indicator relatively sensitive to treatment, but the variation in results surrounding its measurement is widespread within and across studies. This is likely driven by deeper-level heterogeneity factors such as patient baseline characteristics, disease severity, or insufficiently reported intervention details, rather than being caused by a single outlier study.

#### 4.2.3 Tier 4 & 5: the illusion of simple linearity and the challenge of ‘dose’

The fourth and fifth tiers of analysis shifted our discussion from methodological flaws to the core complexity of the intervention itself. The fourth-tier analysis focusing on Astragalus dose yielded a key and enlightening finding: although dose-grouped analysis showed a larger effect size in the medium-dose group (30-90g, e.g., (10)), dose-effect meta-regression (linear and quadratic models) showed no statistical significance (P > 0.40). This clearly suggests that efficacy is not determined by Astragalus dose alone in a simple, linear fashion. The extreme heterogeneity of efficacy within the high-dose group (I² = 93.3%, even under a random-effects model)—ranging from significant improvement in (9) to no effect in (8)—cannot be explained by the dose itself, suggesting that unmeasured factors such as patient pattern differentiation or herb compatibility play a critical role.

The absence of a simple linear dose-response invites speculation about more complex relationships. One hypothesis, consistent with TCM theory, is that efficacy depends on the ‘match’ between intervention details (e.g., Astragalus dose and herb compatibility) and the patient’s specific pattern (‘pattern-syndrome correspondence’) (16, 27). However, this remains a hypothesis generated from observed data patterns. Baseline disease severity (initial NCV) and diabetes duration were not uniformly reported. We cannot rule out that these unadjusted confounders, rather than TCM pattern per se, contributed to the lack of efficacy in high-dose studies. We cannot rule out that these factors, rather than TCM pattern per se, are the primary drivers of the observed variation in treatment response. Therefore, the ‘pattern-syndrome correspondence’ explanation is proposed as a plausible yet unverified framework for future hypothesis-driven research.

The descriptive evidence matrix from the fifth tier provides further support. The data indicate that “BYHWD combined with ALA” in clinical research is a highly heterogeneous composite intervention. Its efficacy may be the result of the complex interaction of multiple factors, including Astragalus dose, patient TCM pattern (e.g., whether it is “severe qi deficiency and marked blood stasis”), and the compatibility of blood-activating and collateral-unblocking herbs within the formula (e.g., whether insect drugs are added). For example, low-dose Astragalus combined with scorpion and centipede (13) showed efficacy, possibly because the “searching wind and unblocking collaterals” power of the insect drugs compensated for insufficient qi-tonifying. Conversely, high-dose Astragalus was effective in patients with long disease duration and evident blood stasis (9) but could be ineffective when the pattern was mismatched or compatibility was inappropriate (8). Modern pharmacological research corroborates this: BYHWD with high-dose Astragalus exhibits stronger neuroprotective effects in DPN model rats, with mechanisms closely related to upregulating SIRT1 and inhibiting the p53-mediated mitochondrial apoptosis pathway (17, 28). Therefore, the analytical results of this study provide a modern empirical annotation for the traditional theory of “pattern-syndrome correspondence” from the perspective of clinical data: maximizing efficacy critically depends on the precise matching of the intervention (herbal dose and compatibility) with the patient’s specific pathological state (pattern). Furthermore, this analysis underscores a fundamental limitation in much of herbal medicine clinical research: the use of crude herb weight (grams) as the sole measure of ‘dose.’ Although we detailed the botanical composition in **Supplementary Material S4**, strict chemical standardization was lacking. The pharmacological activity of an herb is determined by its specific bioactive constituents (e.g., astragaloside IV, calycosin in Astragalus), whose concentrations vary substantially based on source, processing, and preparation. Our dose-related findings, therefore, pertain to a crude and noisy proxy variable. Future definitive studies must advance to quantitative standardization based on characterized chemical markers to reduce this major source of intervention-related heterogeneity.

#### 4.2.4 Allochrony of symptom improvement and neural repair: significance of different outcome measures

The significant improvement in overall response rate and TCM syndrome scores observed in this study, contrasted with the high heterogeneity and uncertain improvement in NCV indicators, may reflect the allochrony of repair across different pathological levels of DPN and the differing abilities of various assessment tools to capture changes in different dimensions. The combination therapy may rapidly alleviate subjective symptoms such as numbness, pain, and fatigue through mechanisms like reducing oxidative stress (18, 29), improving endometrial microcirculation (19, 30), and modulating pain signal transduction. These changes are captured by the overall response rate and syndrome scores. However, the axonal structural integrity or myelin repair of large myelinated fibers, as reflected by NCV, may be a slower process requiring longer treatment durations to become evident (20, 31). A recent meta-analysis on ALA also reported a similar phenomenon, namely that its improvement of symptom scores was superior to its improvement of nerve conduction velocity (2). This “symptom-electrophysiology dissociation” reminds us that patient-reported outcomes (PROs) and objective physiological indicators hold equally important clinical value; they should be considered complementary, not substitutive, dimensions of efficacy assessment. In clinical practice, staged, multi-dimensional treatment expectations should be established for patients accordingly.

### 4.3 Clinical implications: a cautious interpretation based on current evidence

Given the ‘add-on’ design of all included RCTs (ALA+BYHWD vs. ALA alone), the observed benefits of the combination can be definitively described as an additive or complementary effect. We avoid the term ‘pharmacological synergy’ as our study design (A+B vs A) cannot isolate the independent contribution of BYHWD. The mechanisms of ALA (direct antioxidant) and BYHWD (multi-target regulation) are theoretically complementary. Thus, the combination represents a promising therapeutic strategy grounded in mechanism-informed rationale, whose clinical superiority is supported by existing, albeit limited, evidence.

### 4.4 A roadmap for future research: from empirical trials to standardized, precision inquiry

Based on the above findings, we propose the following actionable recommendations:

Implications for Clinical Practice: This combination regimen is a safe and effective option for improving subjective symptoms in DPN patients, particularly suitable for those whose TCM pattern is identified as qi deficiency and blood stasis. When prescribing, emphasis should be placed on TCM pattern differentiation. The Astragalus dose need not be blindly increased; the classic dose around 60g may be a safer, more universally applicable starting point. However, for patients clearly identified with the pattern of “severe qi deficiency and marked blood stasis” who have long-term, severe conditions, and referring to basic research evidence and effective cases from this meta-analysis (17), the cautious use of high-dose Astragalus (e.g., 90-120g) may yield better effects targeting neural structural repair, provided the compatibility of blood-activating and collateral-unblocking herbs is appropriate. Patients should be informed that symptom improvement may precede and be greater than objective recovery in neuroelectrophysiology, setting reasonable treatment expectations.

Recommendations for Future Research Paradigms: A fundamental paradigm shift is necessary in this field of research.

1. Mandatory Methodological Standardization and Diversification of Outcome Measures: Standardized measurement protocols for core objective outcome indicators (e.g., common peroneal nerve MCV) must be unified and pre-registered. Simultaneously, drawing on international consensus, more sensitive and specific assessment tool combinations should be adopted, such as combining traditional nerve conduction velocity (assessing large fibers), corneal confocal microscopy (non-invasively assessing small fibers), and quantitative sensory testing, to comprehensively and stratify the assessment of repair across different nerve fiber types (1).
2. Transparent and Precise Intervention Reporting: Strict adherence to reporting guidelines for Chinese herbal medicine clinical trials (e.g., CONSORT-CHM) is required to fully report all details of the herbal prescription (dose of each herb, origin, decoction and administration method). Future study designs should proactively incorporate TCM pattern classification and core herb doses (especially the sovereign herb Astragalus) as key variables for stratification or subgroup analysis in prospective designs to explore precise matching relationships.
3. Deepening Research Design: From “Verifying Efficacy” to “Exploring Matching”: Future trials should move beyond the current limitations of small sample sizes, short durations (mostly 4-8 weeks), and reliance on subjective response rates. They should shift towards conducting large-sample, long-duration (≥6 months), multi-center pragmatic comparative effectiveness research or precision medicine trials based on biomarkers (e.g., oxidative stress, inflammatory markers) or precise TCM pattern stratification. This is essential to confirm long-term neuroprotective effects and disease-modifying potential (21, 34).

### 4.5 Limitations and strengths

This study has several important limitations, many of which reflect the state of the primary research it synthesizes: 1) The high risk of bias in included trials (e.g., lack of blinding) limits confidence in the estimates. 2) Our analysis is constrained by the poor reporting of key covariates (e.g., baseline disease severity) in original studies, restricting causal exploration. 3) As discussed, the use of crude herb weight is a major limitation. 4) The exclusive inclusion of Chinese studies may limit the generalizability of findings to other healthcare contexts and raises the possibility of region-specific bias.

Strengths: The primary strength is the innovative application of a pre-specified, multi-tiered analytical framework to transparently deconstruct heterogeneity rather than obscure it. This transforms a methodological challenge into the central finding, providing a replicable model for evaluating complex interventions and a clear agenda for methodological reform in the field. However, the strengths of this study are significant and innovative: This is the first systematic review and meta-analysis of the specific regimen of BYHWD combined with ALA. We innovatively employed a five-tiered progressive framework to deeply deconstruct the high-heterogeneity evidence, integrating the TCM theory of “pattern-syndrome correspondence” with modern clinical research heterogeneity analysis methodology. This approach not only yielded clinical conclusions regarding efficacy and safety but also profoundly exposed key methodological shortcomings in current DPN research involving Chinese medicine, particularly regarding objective indicator measurement and reporting of complex interventions. It provides a clear roadmap for standardization and precision in future efforts to construct a more rigorous, evaluable efficacy evidence system for integrated Chinese and Western medicine that aligns with the intrinsic principles of TCM.

## 5 Conclusion

In summary, this systematic review indicates that BYHWD-ALA combination therapy may improve subjective symptoms of DPN, but the evidence for objective neurological improvement is currently inconclusive due to fundamental methodological heterogeneity. The principal contribution of this work lies in its methodological approach: by rigorously deconstructing the sources of inconsistency, it moves beyond a simple efficacy assessment to provide a critical appraisal of the existing evidence base and a concrete roadmap for designing more rigorous, standardized, and informative clinical trials of integrated Chinese herbal medicine.
