# Supplementary material for: Efficacy and safety of Buyang Huanwu Decoction combined with α-lipoic acid for diabetic peripheral neuropathy: a systematic review with in-depth heterogeneity deconstruction and methodological appraisal
Source: Front Endocrinol (Lausanne). 2026 Apr 30;17:1778262. doi: 10.3389/fendo.2026.1778262 (PMC13171351; doi:10.3389/fendo.2026.1778262)
Supplement: Supplementary file 1 [file DataSheet1.docx]

Supplementary Material

**Contents Page**

[1 Supplementary Material S1. PRISMA 2020 checklist	2](#_Toc833907803)

[2 Supplementary Material S2. Database and Search Strategies	8](#_Toc1010164699)

[2.1 PubMed	8](#_Toc1979866558)

[2.2 Embase	9](#_Toc358130041)

[2.3 Cochrane	10](#_Toc1842420193)

[2.4 WOS	11](#_Toc989477658)

[2.5 CNKI	12](#_Toc37635638)

[2.6 Wan Fang	13](#_Toc1181975648)

[2.7 VIP	13](#_Toc1240981186)

[2.8 CBM	14](#_Toc809613438)

[2.9 ClinicalTrials.gov	15](#_Toc716665074)

[2.10 Chinese Clinical Trial Registry	15](#_Toc1901606342)

[3 Supplementary Material S3. The procedure for preliminary screening of the literature	16](#_Toc1446155340)

[4 Supplementary Material S4. Botanical Information of Buyang Huanwu Decoction Composition	16](#_Toc312882634)

[5 Supplementary Material S5. Subgroup analysis images	18](#_Toc1578461782)

[6 Supplementary Material S6. Sensitivity analysis	21](#_Toc1341678683)

[7 Supplementary Material S7 . Assessment of evidence quality for each outcome	30](#_Toc1015331681)

# 1 Supplementary Material S1. PRISMA 2020 checklist

| **Section and Topic** | **Item #** | **Checklist item** | **Location where item is reported** |
| --- | --- | --- | --- |
| **TITLE** | | |  |
| Title | 1 | Identify the report as a systematic review. |  |
| **ABSTRACT** | | |  |
| Abstract | 2 | See the PRISMA 2020 for Abstracts checklist. | *Abstract section* |
| **INTRODUCTION** | | |  |
| Rationale | 3 | Describe the rationale for the review in the context of existing knowledge. | - |
| Objectives | 4 | Provide an explicit statement of the objective(s) or question(s) the review addresses. | - |
| **METHODS** | | |  |
| Eligibility criteria | 5 | Specify the inclusion and exclusion criteria for the review and how studies were grouped for the syntheses. | - |
| Information sources | 6 | Specify all databases, registers, websites, organisations, reference lists and other sources searched or consulted to identify studies. Specify the date when each source was last searched or consulted. | ***(search date: December 2025)*** |
| Search strategy | 7 | Present the full search strategies for all databases, registers and websites, including any filters and limits used. | Supplementary Material S2 |
| Selection process | 8 | Specify the methods used to decide whether a study met the inclusion criteria of the review, including how many reviewers screened each record and each report retrieved, whether they worked independently, and if applicable, details of automation tools used in the process. | - |
| Data collection process | 9 | Specify the methods used to collect data from reports, including how many reviewers collected data from each report, whether they worked independently, any processes for obtaining or confirming data from study investigators, and if applicable, details of automation tools used in the process. | - |
| Data items | 10a | List and define all outcomes for which data were sought. Specify whether all results that were compatible with each outcome domain in each study were sought (e.g. for all measures, time points, analyses), and if not, the methods used to decide which results to collect. | - |
|  | 10b | List and define all other variables for which data were sought (e.g. participant and intervention characteristics, funding sources). Describe any assumptions made about any missing or unclear information. | - |
| Study risk of bias assessment | 11 | Specify the methods used to assess risk of bias in the included studies, including details of the tool(s) used, how many reviewers assessed each study and whether they worked independently, and if applicable, details of automation tools used in the process. | - |
| Effect measures | 12 | Specify for each outcome the effect measure(s) (e.g. risk ratio, mean difference) used in the synthesis or presentation of results. | - |
| Synthesis methods | 13a | Describe the processes used to decide which studies were eligible for each synthesis (e.g. tabulating the study intervention characteristics and comparing against the planned groups for each synthesis (item #5)). | - |
|  | 13b | Describe any methods required to prepare the data for presentation or synthesis, such as handling of missing summary statistics, or data conversions. | - |
|  | 13c | Describe any methods used to tabulate or visually display results of individual studies and syntheses. | - |
|  | 13d | Describe any methods used to synthesize results and provide a rationale for the choice(s). If meta-analysis was performed, describe the model(s), method(s) to identify the presence and extent of statistical heterogeneity, and software package(s) used. | - |
|  | 13e | Describe any methods used to explore possible causes of heterogeneity among study results (e.g. subgroup analysis, meta-regression). | - |
|  | 13f | Describe any sensitivity analyses conducted to assess robustness of the synthesized results. | - |
| Reporting bias assessment | 14 | Describe any methods used to assess risk of bias due to missing results in a synthesis (arising from reporting biases). | - |
| Certainty assessment | 15 | Describe any methods used to assess certainty (or confidence) in the body of evidence for an outcome. | - |
| **RESULTS** | | |  |
| Study selection | 16a | Describe the results of the search and selection process, from the number of records identified in the search to the number of studies included in the review, ideally using a flow diagram. | *Figure 2* |
|  | 16b | Cite studies that might appear to meet the inclusion criteria, but which were excluded, and explain why they were excluded. | *Supplementary Material S11* |
| Study characteristics | 17 | Cite each included study and present its characteristics. | *Table 1, Supplementary Material S5* |
| Risk of bias in studies | 18 | Present assessments of risk of bias for each included study. | Figure 3 |
| Results of individual studies | 19 | For all outcomes, present, for each study: (a) summary statistics for each group (where appropriate) and (b) an effect estimate and its precision (e.g. confidence/credible interval), ideally using structured tables or plots. | - |
| Results of syntheses | 20a | For each synthesis, briefly summarise the characteristics and risk of bias among contributing studies. | - |
|  | 20b | Present results of all statistical syntheses conducted. If meta-analysis was done, present for each the summary estimate and its precision (e.g. confidence/credible interval) and measures of statistical heterogeneity. If comparing groups, describe the direction of the effect. | - |
|  | 20c | Present results of all investigations of possible causes of heterogeneity among study results. | - |
|  | 20d | Present results of all sensitivity analyses conducted to assess the robustness of the synthesized results. | Table 4 |
| Reporting biases | 21 | Present assessments of risk of bias due to missing results (arising from reporting biases) for each synthesis assessed. | - |
| Certainty of evidence | 22 | Present assessments of certainty (or confidence) in the body of evidence for each outcome assessed. | P11, Supplementary Material S11 |
| **DISCUSSION** | | |  |
| Discussion | 23a | Provide a general interpretation of the results in the context of other evidence. | - |
|  | 23b | Discuss any limitations of the evidence included in the review. | - |
|  | 23c | Discuss any limitations of the review processes used. | - |
|  | 23d | Discuss implications of the results for practice, policy, and future research. | - |
| **OTHER INFORMATION** | | |  |
| Registration and protocol | 24a | Provide registration information for the review, including register name and registration number, or state that the review was not registered. | *P4, CRD420251267606* |
|  | 24b | Indicate where the review protocol can be accessed, or state that a protocol was not prepared. | - |
|  | 24c | Describe and explain any amendments to information provided at registration or in the protocol. | - |
| Support | 25 | Describe sources of financial or non-financial support for the review, and the role of the funders or sponsors in the review. | - |
| Competing interests | 26 | Declare any competing interests of review authors. | - |
| Availability of data, code and other materials | 27 | Report which of the following are publicly available and where they can be found: template data collection forms; data extracted from included studies; data used for all analyses; analytic code; any other materials used in the review. | - |

*From:* Page MJ, McKenzie JE, Bossuyt PM, Boutron I, Hoffmann TC, Mulrow CD, et al. The PRISMA 2020 statement: an updated guideline for reporting systematic reviews. BMJ 2021;372:n71. doi: 10.1136/bmj.n71

# 2 Supplementary Material S2. Database and Search Strategies

## 2.1 PubMed

The retrieval of the PubMed database was conducted on **December 17**, 2025, and no records were retrieved.

| Search | Query | Results |
| --- | --- | --- |
| #6 | ((#3) AND (#4)) AND (#5) | 0 |
| #5 | "randomized controlled trial"[Publication Type] OR "controlled clinical trial"[Publication Type] OR "randomized"[Title/Abstract] OR "placebo"[Title/Abstract] OR "clinical trials as topic"[MeSH Terms] OR "randomly"[Title/Abstract] OR "trial"[Title] | 429911 |
| #4 | (("Buyang Huanwu Decoction"[Title/Abstract] OR "Buyang Huanwu Tang"[Title/Abstract])) AND ("alpha-lipoic acid"[Title/Abstract] OR "alpha-lipoic acid"[Title/Abstract] OR "thioctic acid"[Title/Abstract] OR "lipoic acid"[Title/Abstract] OR "ALA"[Title/Abstract] OR "Thioctacid"[Title/Abstract]) | 0 |
| #3 | (#1) OR (#2) | 10282 |
| #2 | "Diabetic Peripheral Neuropathy"[Title/Abstract] OR "Diabetic Peripheral Neuropathies"[Title/Abstract] OR "Diabetic Polyneuropathy"[Title/Abstract] OR "Diabetic Polyneuropathies"[Title/Abstract] OR "Peripheral Diabetic Neuropathy"[Title/Abstract] OR "DPN"[Title/Abstract] OR "Painful Diabetic Neuropathy"[Title/Abstract] OR "Painful Diabetic Neuropathies"[Title/Abstract] OR "Diabetic Foot"[Title/Abstract] OR "Diabetic distal symmetric polyneuropathy"[Title/Abstract] | 8734 |
| #1 | "Diabetic Neuropathies"[Mesh] | 6323 |

## 2.2 Embase

The retrieval of the Embase database was conducted on December 17, 2025, and no records were retrieved.

| Search |  | Results |
| --- | --- | --- |
| #7 | #3 AND #4 AND #5 AND #6 | 0 |
| #6 | 'randomized controlled trial'/exp OR 'randomized controlled trial':ti,ab,kw OR 'rct':ti,ab,kw OR 'randomized':ti,ab,kw OR 'randomly':ti,ab,kw OR 'placebo':ti,ab,kw OR 'clinical trial':ti,ab,kw | 2,496,392 |
| #5 | 'alpha-lipoic acid':ti,ab,kw OR 'α-lipoic acid':ti,ab,kw OR 'thioctic acid':ti,ab,kw OR 'lipoic acid':ti,ab,kw OR 'ala':ti,ab,kw OR 'thioctacid':ti,ab,kw | 66,700 |
| #4 | 'buyang huanwu decoction':ti,ab,kw OR 'buyang huanwu tang':ti,ab,kw OR 'tonifying yang to restore five decoction':ti,ab,kw | 458 |
| #3 | #1 OR #2 | 61,847 |
| #2 | 'diabetic peripheral neuropathy':ti,ab,kw OR 'diabetic peripheral neuropathies':ti,ab,kw OR 'diabetic polyneuropathy':ti,ab,kw OR 'diabetic polyneuropathies':ti,ab,kw OR 'peripheral diabetic neuropathy':ti,ab,kw OR 'dpn':ti,ab,kw OR 'painful diabetic neuropathy':ti,ab,kw OR 'painful diabetic neuropathies':ti,ab,kw OR 'diabetic foot':ti,ab,kw OR 'distal symmetric polyneuropathy':ti,ab,kw | 35,333 |
| #1 | 'diabetic neuropathy'/exp OR 'diabetic neuropathy' | 38,087 |

## 2.3 Cochrane

The retrieval of the Cochrane Library was conducted on December 17, 2025, and no records were retrieved.

| Search |  | Results |
| --- | --- | --- |
| #6 | #3 AND #4 AND #5 | 0 |
| #5 | 'alpha-lipoic acid':ti,ab,kw OR 'α-lipoic acid':ti,ab,kw OR 'thioctic acid':ti,ab,kw OR 'lipoic acid':ti,ab,kw OR 'ala':ti,ab,kw OR 'thioctacid':ti,ab,kw | 2503 |
| #4 | 'buyang huanwu decoction':ti,ab,kw OR 'buyang huanwu tang':ti,ab,kw OR 'tonifying yang to restore five decoction':ti,ab,kw | 101 |
| #3 | #1 OR #2 | 10097 |
| #2 | 'diabetic peripheral neuropathy':ti,ab,kw OR 'diabetic peripheral neuropathies':ti,ab,kw OR 'diabetic polyneuropathy':ti,ab,kw OR 'diabetic polyneuropathies':ti,ab,kw OR 'peripheral diabetic neuropathy':ti,ab,kw OR 'dpn':ti,ab,kw OR 'painful diabetic neuropathy':ti,ab,kw OR 'painful diabetic neuropathies':ti,ab,kw OR 'diabetic foot':ti,ab,kw OR 'distal symmetric polyneuropathy':ti,ab,kw | 8091 |
| #1 | MeSH descriptor: [Diabetic Nephropathies] explode all trees | 2053 |

## 2.4 WOS

The retrieval of the WOS database was conducted on December 17, 2025, and no records were retrieved.

| History | Search Query | Results |
| --- | --- | --- |
| #7 | #6 AND #5 AND #4 AND #3 | 0 |
| #6 | TS=(("randomized controlled trial" OR "randomised controlled trial" OR RCT OR randomly OR randomized OR randomised OR placebo OR trial OR "clinical trial" OR "controlled clinical trial")) | 1828502 |
| #5 | TS=(("alpha-lipoic acid" OR "α-lipoic acid" OR "thioctic acid" OR "lipoic acid" OR ALA OR Thioctacid)) | 30916 |
| #4 | ((TS=(diabetic peripheral neuropathy)) AND TS=(("diabetic peripheral neuropathies" OR "diabetic polyneuropathy" OR "diabetic polyneuropathies" OR "peripheral diabetic neuropathy" OR DPN OR "painful diabetic neuropathy" OR "painful diabetic neuropathies" OR "diabetic foot" OR "distal symmetric polyneuropathy"))) AND TS=("Buyang Huanwu Decoction" OR "Buyang Huanwu Tang" OR "Tonifying Yang to Restore Five Decoction") | 1 |
| #3 | #2 OR #1 | 7910 |
| #2 | (TS=(diabetic peripheral neuropathy)) AND TS=(("diabetic peripheral neuropathies" OR "diabetic polyneuropathy" OR "diabetic polyneuropathies" OR "peripheral diabetic neuropathy" OR DPN OR "painful diabetic neuropathy" OR "painful diabetic neuropathies" OR "diabetic foot" OR "distal symmetric polyneuropathy")) | 3553 |
| #1 | TS=(diabetic peripheral neuropathy) | 7910 |

## 2.5 CNKI

The retrieval of the CNKI database was conducted on December 17, 2025, and a total of 28 records were retrieved.

SU=('糖尿病周围神经病变' + '糖尿病性周围神经病变' + '糖尿病神经病变' + '糖尿病性神经病' + '糖尿病神经病' + '糖尿病性多发性周围神经病' + '远端对称性多发性神经病变' + '痛性糖尿病周围神经病变' + 'DPN' + '消渴病痹证') AND SU=('α-硫辛酸' + '硫辛酸' + '奥力宝' + '凯抒汀' + '健肝灵' + 'DL-硫辛酸' + '二硫辛酸') AND SU=('补阳还五汤' + '补阳还五' )

## 2.6 Wan Fang

The retrieval of the Wan Fang database was conducted on December 17, 2025, and a total of 40 records were retrieved.

**(主题:(糖尿病周围神经病变 OR 糖尿病性周围神经病变 OR 糖尿病神经病变 OR 糖尿病性神经病 OR 糖尿病神经病 OR 糖尿病性多发性周围神经病 OR 远端对称性多发性神经病变 OR 痛性糖尿病周围神经病变 OR DPN OR 消渴病痹证) OR 题名或关键词:(糖尿病周围神经病变 OR 糖尿病性周围神经病变 OR 糖尿病神经病变 OR 糖尿病性神经病 OR 糖尿病神经病 OR 糖尿病性多发性周围神经病 OR 远端对称性多发性神经病变 OR 痛性糖尿病周围神经病变 OR DPN OR 消渴病痹证)) AND (主题:(α-硫辛酸 OR 硫辛酸 OR 奥力宝 OR 凯抒汀 OR 健肝灵 OR DL-硫辛酸 OR 二硫辛酸) OR 题名或关键词:(α-硫辛酸 OR 硫辛酸 OR 奥力宝 OR 凯抒汀 OR 健肝灵 OR DL-硫辛酸 OR 二硫辛酸)) AND (主题:(补阳还五汤 OR 补阳还五) OR 题名或关键词:(补阳还五汤 OR 补阳还五))**

## 2.7 VIP

The retrieval of the VIP database was conducted on December 17, 2025, and a total of 30 records were retrieved.

**((M=(糖尿病周围神经病变+糖尿病性周围神经病变+糖尿病神经病变+糖尿病性神经病+糖尿病神经病+糖尿病性多发性周围神经病+远端对称性多发性神经病变+痛性糖尿病周围神经病变+DPN+消渴病痹证)) OR (R=(糖尿病周围神经病变+糖尿病性周围神经病变+糖尿病神经病变+糖尿病性神经病+糖尿病神经病+糖尿病性多发性周围神经病+远端对称性多发性神经病变+痛性糖尿病周围神经病变+DPN+消渴病痹证))) AND ((M=(α-硫辛酸+硫辛酸+奥力宝+凯抒汀+健肝灵+DL-硫辛酸+二硫辛酸)) OR (R=(α-硫辛酸+硫辛酸+奥力宝+凯抒汀+健肝灵+DL-硫辛酸+二硫辛酸))) AND ((M=(补阳还五汤+补阳还五)) OR (R=(补阳还五汤+补阳还五))**

## 2.8 CBM

The retrieval of the CBM database was conducted on December 17, 2025, and a total of 16 records were retrieved.

**(("糖尿病周围神经病变"[不加权:扩展]) OR ("糖尿病性周围神经病变"[常用字段:智能] OR "糖尿病神经病变"[常用字段:智能] OR "糖尿病性神经病"[常用字段:智能] OR "糖尿病神经病"[常用字段:智能] OR "糖尿病性多发性周围神经病"[常用字段:智能] OR "远端对称性多发性神经病变"[常用字段:智能] OR "痛性糖尿病周围神经病变"[常用字段:智能] OR "DPN"[常用字段:智能] OR "消渴病痹证"[常用字段:智能])) AND (("α-硫辛酸"[不加权:扩展] OR "硫辛酸"[不加权:扩展] OR "奥力宝"[不加权:扩展] OR "凯抒汀"[常用字段:智能] OR "健肝灵"[常用字段:智能] OR "DL-硫辛酸"[常用字段:智能] OR "二硫辛酸"[常用字段:智能])) AND (("补阳还五汤"[不加权:扩展] OR "补阳还五"[不加权:扩展]))**

## 2.9 ClinicalTrials.gov

The retrieval of the ClinicalTrials.gov Registry was conducted on December 17, 2025, and no records were retrieved.

## 2.10 Chinese Clinical Trial Registry

The retrieval of the Chinese Clinical Trial Registry was conducted on December 17, 2025 and no records were retrieved.

# 3 Supplementary Material S3. The procedure for preliminary screening of the literature

# 4 Supplementary Material S4. Botanical Information of Buyang Huanwu Decoction Composition

| **Chinese Name** | **Latin Name (Full with Authority)** | **Plant Family** | **Part Used** | **Reference Dosage in Classic Formula (g)** |
| --- | --- | --- | --- | --- |
| Milkvetch Root (Raw) Huang Qi (Sheng) | Astragalus mongholicus Bunge or Astragalus membranaceus (Fisch.) Bunge | Fabaceae | Root | 120 |
| Chinese Angelica Tail Dang Gui Wei | Angelica sinensis (Oliv.) Diels | Apiaceae | Root Tail (Specialized part) | 6 |
| Red Peony Root Chi Shao | Paeonia lactiflora Pall. or Paeonia veitchii Lynch | Paeoniaceae | Root (Peeled) | 5 |
| Szechuan Lovage Rhizome Chuan Xiong | Ligusticum chuanxiong Hort. | Apiaceae | Rhizome | 3 |
| Peach Kernel Tao Ren | Prunus persica (L.) Batsch or Prunus davidiana (Carrière) Franch. | Rosaceae | Seed | 3 |
| Safflower Hong Hua | Carthamus tinctorius L. | Asteraceae | Flower | 3 |
| Earthworm Di Long | Pheretima aspergillum (E. Perrier) or Pheretima vulgaris Chen | Megascolecidae (Animal) | Dried Body (Earthworm) | 3 |
| **Notes:**  **Classical Formula Origin:** This table is based on the classic formula of Buyang Huanwu Decoction as recorded in "Corrections of Errors in Medical Classics" (Yi Lin Gai Cuo) by Wang Qingren in the Qing Dynasty. In the clinical studies included in your systematic review, researchers often modify the formula by adding or subtracting herbs (e.g., adding Achyranthes root, Salvia root, Scorpion, etc.) based on the specific manifestations of the "Qi Deficiency and Blood Stasis" pattern. Therefore, it is essential to verify the actual composition against the prescriptions documented in each extracted study.  **Dosage of Milkvetch Root (Astragalus):** In the original formula, Milkvetch Root is the sovereign herb with a uniquely large dosage (120g). However, in clinical practice, the dosage often starts at 30-60g and is gradually increased based on therapeutic effect. The variation in dosage across different studies may be a significant point for analysis.  **Characteristic Chemical Constituents:** Modern pharmacological research has identified various active components in Buyang Huanwu Decoction, such as **Calycosin-glucoside** and **Calycosin** from Milkvetch Root, **Paeoniflorin** from Red Peony Root, and **Hydroxysafflor yellow A** from Safflower. These components are often used as markers for quality control. | | | | |

# 5 Supplementary Material S5. Subgroup analysis images

# 6 Supplementary Material S6. Sensitivity analysis

| Table: Complete Results of Leave-One-Out Sensitivity Analyses for All Nerve Conduction Velocity Outcomes | | | | | | | | |
| --- | --- | --- | --- | --- | --- | --- | --- | --- |
| **Outcome** | **No. of Studies Included** | **Primary Pooled Effect Size (95% CI)** | **Primary I² (%)** | **Study Omitted** | **Effect Size After Omission (95% CI)** | **I² After Omission (%)** | **P for Subgroup Difference After Omission (if applicable)** | **Robustness Conclusion** |
| Composite MCV | 10 | SMD = 1.85 [1.08, 2.62] | 96 | guo2015 | SMD = 2.00 [1.16, 2.85] | 97 | Not applicable | The confidence interval after omission does not overlap with the primary result. Study 1 has a moderate influence on the pooled effect, but the overall effect remains significant. |
| Composite MCV | 10 | SMD = 1.85 [1.08, 2.62] | 96 | jin2020 | SMD = 1.07 [0.56, 1.59] | 92 | Not applicable | The SMD decreased substantially compared to the primary result. Study 2 is a major influencing factor, but the effect remains statistically significant. |
| Composite MCV | 10 | SMD = 1.85 [1.08, 2.62] | 96 | lin2019 | SMD = 1.86 [1.03, 2.70] | 97 | Not applicable | The SMD after omission is highly consistent with the primary result. Study 3 has no significant impact on the pooled effect, indicating good stability. |
| Composite MCV | 10 | SMD = 1.85 [1.08, 2.62] | 96 | liu2015 | SMD = 1.99 [1.10, 2.87] | 97 | Not applicable | The SMD after omission shows minimal difference from the primary result. Study 4 has a slight influence on the pooled effect, and the result is robust. |
| Composite MCV | 10 | SMD = 1.85 [1.08, 2.62] | 96 | liu2017 | SMD = 2.06 [1.25, 2.87] | 96 | Not applicable | The SMD after omission is slightly higher than the primary result, with no essential difference. Study 5 has a minor influence on the pooled effect. |
| Composite MCV | 10 | SMD = 1.85 [1.08, 2.62] | 96 | lu2016 | SMD = 1.82 [1.01, 2.64] | 97 | Not applicable | The SMD after omission is almost identical to the primary result. Study 6 has no impact on the pooled effect, indicating excellent stability. |
| Composite MCV | 10 | SMD = 1.85 [1.08, 2.62] | 96 | song2017 | SMD = 2.01 [1.16, 2.85] | 97 | Not applicable | The SMD after omission shows a minimal difference from the primary result. Study 7 has no significant interference on the pooled effect, and the result is robust. |
| Composite MCV | 10 | SMD = 1.85 [1.08, 2.62] | 96 | yao2016 | SMD = 2.05 [1.21, 2.88] | 97 | Not applicable | The SMD after omission is slightly higher than the primary result, with no substantial difference. Study 8 has a slight influence on the pooled effect. |
| Composite MCV | 10 | SMD = 1.85 [1.08, 2.62] | 96 | zhang2018 | SMD = 1.84 [1.01, 2.67] | 96 | Not applicable | The SMD after omission is highly consistent with the primary result. Study 9 has no impact on the pooled effect, indicating good stability. |
| Composite MCV | 10 | SMD = 1.85 [1.08, 2.62] | 96 | zhao2019 | SMD = 1.89 [1.05, 2.73] | 97 | Not applicable | The SMD after omission shows an extremely minimal difference from the primary result. Study 10 has no significant impact on the pooled effect, and the result is robust. |
| Composite MCV | 10 | SMD = 1.85 [1.08, 2.62] | 96 | zu2020 | SMD = 2.04 [1.18, 2.90] | 97 | Not applicable | The SMD after omission shows no substantial difference from the primary result. Study 11 has a minor influence on the pooled effect, and the result is stable. |
| Composite SCV | 10 | SMD = 1.85 [1.08, 2.62] | 96 | guo2015 | SMD = 2.00 [1.16, 2.85] | 97 | Not applicable | The confidence interval after omission does not overlap with the primary result. This study has a moderate influence on the pooled effect, but the overall effect remains significant. |
| Composite SCV | 10 | SMD = 1.85 [1.08, 2.62] | 96 | jin2020 | SMD = 1.07 [0.56, 1.59] | 92 | Not applicable | The SMD after omission decreased substantially compared to the primary result. This study is a major influencing factor, but the effect remains statistically significant. |
| Composite SCV | 10 | SMD = 1.85 [1.08, 2.62] | 96 | lin2019 | SMD = 1.86 [1.03, 2.70] | 97 | Not applicable | The SMD after omission is highly consistent with the primary result. This study has no significant impact on the pooled effect, indicating good stability. |
| Composite SCV | 10 | SMD = 1.85 [1.08, 2.62] | 96 | liu2015 | SMD = 1.99 [1.10, 2.87] | 97 | Not applicable | The SMD after omission shows minimal difference from the primary result. This study has a slight influence on the pooled effect, and the result is robust. |
| Composite SCV | 10 | SMD = 1.85 [1.08, 2.62] | 96 | liu2017 | SMD = 2.06 [1.25, 2.87] | 96 | Not applicable | The SMD after omission is slightly higher than the primary result, with no essential difference. This study has a minor influence on the pooled effect. |
| Composite SCV | 10 | SMD = 1.85 [1.08, 2.62] | 96 | lu2016 | SMD = 1.82 [1.01, 2.64] | 97 | Not applicable | The SMD after omission is almost identical to the primary result. This study has no impact on the pooled effect, indicating excellent stability. |
| Composite SCV | 10 | SMD = 1.85 [1.08, 2.62] | 96 | song2017 | SMD = 2.01 [1.16, 2.85] | 97 | Not applicable | The SMD after omission shows a minimal difference from the primary result. This study has no significant interference on the pooled effect, and the result is robust. |
| Composite SCV | 10 | SMD = 1.85 [1.08, 2.62] | 96 | yao2016 | SMD = 2.05 [1.21, 2.88] | 97 | Not applicable | The SMD after omission is slightly higher than the primary result, with no substantial difference. This study has a slight influence on the pooled effect. |
| Composite SCV | 10 | SMD = 1.85 [1.08, 2.62] | 96 | zhang2018 | SMD = 1.84 [1.01, 2.67] | 96 | Not applicable | The SMD after omission is highly consistent with the primary result. This study has no impact on the pooled effect, indicating good stability. |
| Composite SCV | 10 | SMD = 1.85 [1.08, 2.62] | 96 | zhao2019 | SMD = 1.89 [1.05, 2.73] | 97 | Not applicable | The SMD after omission shows an extremely minimal difference from the primary result. This study has no significant impact on the pooled effect, and the result is robust. |
| Composite SCV | 10 | SMD = 1.85 [1.08, 2.62] | 96 | zu2020 | SMD = 2.04 [1.18, 2.90] | 97 | Not applicable | The SMD after omission shows no substantial difference from the primary result. This study has a minor influence on the pooled effect, and the result is stable. |
| Common Peroneal Nerve MCV | 8 | MD = 1.01 [0.48, 1.54] | 92 | guo2015 | MD = 1.03 [0.44, 1.62] | 93 | Not applicable | The MD after omission is highly consistent with the primary result. This study has no significant impact on the pooled effect, indicating good stability. |
| Common Peroneal Nerve MCV | 8 | MD = 1.01 [0.48, 1.54] | 92 | lin2019 | MD = 0.89 [0.35, 1.43] | 91 | Not applicable | The MD after omission is slightly lower than the primary result, with overlapping confidence intervals. This study has a slight influence on the pooled effect, and the result is robust. |
| Common Peroneal Nerve MCV | 8 | MD = 1.01 [0.48, 1.54] | 92 | liu2015 (1) | MD = 0.94 [0.36, 1.51] | 92 | Not applicable | The MD after omission shows minimal difference from the primary result. This study has no significant interference on the pooled effect, and the result is stable. |
| Common Peroneal Nerve MCV | 8 | MD = 1.01 [0.48, 1.54] | 92 | liu2015 (2) | MD = 1.18 [0.73, 1.64] | 87 | Not applicable | The MD after omission is slightly higher than the primary result, with overlapping confidence intervals. This study has a minor influence on the pooled effect, and the result is robust. |
| Common Peroneal Nerve MCV | 8 | MD = 1.01 [0.48, 1.54] | 92 | liu2017 | MD = 0.86 [0.33, 1.39] | 91 | Not applicable | The MD after omission is slightly lower than the primary result, with no essential difference. This study has a slight influence on the pooled effect, indicating good stability. |
| Common Peroneal Nerve MCV | 8 | MD = 1.01 [0.48, 1.54] | 92 | lu2016 | MD = 1.06 [0.47, 1.65] | 93 | Not applicable | The MD after omission is highly consistent with the primary result. This study has no impact on the pooled effect, indicating good stability. |
| Common Peroneal Nerve MCV | 8 | MD = 1.01 [0.48, 1.54] | 92 | song2017 (1) | MD = 1.09 [0.51, 1.67] | 92 | Not applicable | The MD after omission shows minimal difference from the primary result. This study has no significant impact on the pooled effect, and the result is robust. |
| Common Peroneal Nerve MCV | 8 | MD = 1.01 [0.48, 1.54] | 92 | song2017 (2) | MD = 0.96 [0.36, 1.55] | 92 | Not applicable | The MD after omission is close to the primary result. This study has a minor influence on the pooled effect, and the result is stable. |
| Common Peroneal Nerve MCV | 8 | MD = 1.01 [0.48, 1.54] | 92 | zu2020 | MD = 1.07 [0.47, 1.67] | 93 | Not applicable | The MD after omission is highly consistent with the primary result. This study has no significant interference on the pooled effect, indicating good stability. |
| Median Nerve SCV | 6 | MD = 2.87 [0.34, 5.39] | 96 | guo2015 | MD = 2.80 [-0.01, 5.60] | 97 | Not applicable | After omission, the MD confidence interval is close to 0, P=0.05, nearing the threshold. This study has a certain influence on the significance of the effect. |
| Median Nerve SCV | 6 | MD = 2.87 [0.34, 5.39] | 96 | liu2015 | MD = 2.39 [-0.61, 5.39] | 97 | Not applicable | After omission, the MD confidence interval contains 0, P=0.12, not statistically significant. This study is a key influencing factor. |
| Median Nerve SCV | 6 | MD = 2.87 [0.34, 5.39] | 96 | liu2017 | MD = 3.40 [0.76, 6.03] | 96 | Not applicable | After omission, the MD is higher than the primary result, P=0.01, significant. This study has a minor influence on the pooled effect, and the result is robust. |
| Median Nerve SCV | 6 | MD = 2.87 [0.34, 5.39] | 96 | song2017 | MD = 2.99 [0.15, 5.82] | 97 | Not applicable | After omission, the MD is close to the primary result, P=0.04, significant. This study has no significant interference on the pooled effect, and the result is stable. |
| Median Nerve SCV | 6 | MD = 2.87 [0.34, 5.39] | 96 | yao2016 | MD = 3.29 [0.56, 6.02] | 97 | Not applicable | After omission, the MD is slightly higher than the primary result, P=0.02, significant. This study has a slight influence on the pooled effect, indicating good stability. |
| Median Nerve SCV | 6 | MD = 2.87 [0.34, 5.39] | 96 | zhang2018 | MD = 1.99 [-0.22, 4.20] | 94 | Not applicable | After omission, the MD confidence interval contains 0, P=0.08, not statistically significant. This study has a significant influence on the effect significance. |
| Median Nerve SCV | 6 | MD = 2.87 [0.34, 5.39] | 96 | zu2020 | MD = 3.20 [0.40, 6.00] | 96 | Not applicable | After omission, the MD is higher than the primary result, P=0.03, significant. This study has no significant impact on the pooled effect, and the result is robust. |
| Median Nerve MCV | 6 | MD = 1.93 [0.03, 3.83] | 97 | guo2015 | MD = 1.94 [-0.15, 4.03] | 97 | Not applicable | After omission, the MD confidence interval contains 0, P=0.07, not statistically significant. This study has a certain influence on the effect significance. |
| Median Nerve MCV | 6 | MD = 1.93 [0.03, 3.83] | 97 | liu2015 | MD = 1.80 [-0.35, 3.95] | 97 | Not applicable | After omission, the MD confidence interval contains 0, P=0.10, not statistically significant. This study is a key influencing factor. |
| Median Nerve MCV | 6 | MD = 1.93 [0.03, 3.83] | 97 | liu2017 | MD = 2.23 [-0.02, 4.49] | 97 | Not applicable | After omission, the MD confidence interval is close to 0, P=0.05, threshold. This study has a significant influence on the effect significance. |
| Median Nerve MCV | 6 | MD = 1.93 [0.03, 3.83] | 97 | song2017 | MD = 1.80 [-0.26, 3.87] | 97 | Not applicable | After omission, the MD confidence interval contains 0, P=0.09, not statistically significant. This study has a noticeable influence on the effect significance. |
| Median Nerve MCV | 6 | MD = 1.93 [0.03, 3.83] | 97 | yao2016 | MD = 2.30 [0.10, 4.50] | 97 | Not applicable | After omission, the MD confidence interval does not contain 0, P=0.04, significant. This study has a minor influence on the pooled effect, and the result is robust. |
| Median Nerve MCV | 6 | MD = 1.93 [0.03, 3.83] | 97 | zhang2018 | MD = 1.01 [0.14, 1.88] | 76 | Not applicable | After omission, I² decreased substantially, MD confidence interval does not contain 0, P=0.02, significant. This study is the main source of heterogeneity. |
| Median Nerve MCV | 6 | MD = 1.93 [0.03, 3.83] | 97 | zu2020 | MD = 2.20 [-0.18, 4.59] | 96 | Not applicable | After omission, the MD confidence interval contains 0, P=0.07, not statistically significant. This study has a certain influence on the effect significance. |
| Sural Nerve SCV | 3 | MD = 2.39 [-0.53, 5.30] | 93 | guo2015 | MD = 3.14 [-0.27, 6.55] | 94 | Not applicable | After omission, MD increased slightly, confidence interval is close to 0, P=0.07, threshold. This study has a certain influence on the effect significance. |
| Sural Nerve SCV | 3 | MD = 2.39 [-0.53, 5.30] | 93 | lin2019 | MD = 1.26 [-1.74, 4.26] | 91 | Not applicable | After omission, MD decreased, confidence interval contains 0, P=0.41, not statistically significant. This study is a key influencing factor. |
| Sural Nerve SCV | 3 | MD = 2.39 [-0.53, 5.30] | 93 | lu2016 | MD = 1.82 [-2.29, 5.93] | 95 | Not applicable | After omission, MD decreased, confidence interval contains 0, P=0.39, not statistically significant. This study has a noticeable influence on the effect significance. |
| Sural Nerve SCV | 3 | MD = 2.39 [-0.53, 5.30] | 93 | song2017 | MD = 3.32 [0.38, 6.26] | 91 | Not applicable | After omission, MD increased and confidence interval does not contain 0, P=0.03, significant. This study has a major influence on the pooled effect and is the main reason for the non-significant primary effect. |
| MNCV | 1 | MD = 3.30 [1.19, 5.41] | 98 | jin2020 | MD = 2.21 [1.69, 2.73] | Not applicable | Not applicable | After omission, only the single study zhao2019 remains. MD confidence interval does not contain 0, P<0.001, significant. This study has a certain influence on the pooled effect. |
| MNCV | 1 | MD = 3.30 [1.19, 5.41] | 98 | zhao2019 | MD = 4.36 [4.25, 4.47] | Not applicable | Not applicable | After omission, only the single study jin2020 remains. MD confidence interval does not contain 0, P<0.001, significant. This study has a major influence on the pooled effect, and the primary heterogeneity was extremely high (I²=98%). |
| SNCV | 2 | MD = 2.07 [0.14, 4.01] | 94 | jin2020 | MD = 1.28 [-0.20, 2.76] | 68 | Not applicable | After omission, the MD confidence interval contains 0, P=0.09, not statistically significant. This study has an important influence on the effect significance. |
| SNCV | 2 | MD = 2.07 [0.14, 4.01] | 94 | liu2017 | MD = 2.04 [-0.69, 4.77] | 97 | Not applicable | After omission, the MD confidence interval contains 0, P=0.14, not statistically significant, and heterogeneity remains extremely high. This study is a key influencing factor. |
| SNCV | 2 | MD = 2.07 [0.14, 4.01] | 94 | zhao2019 | MD = 2.95 [1.76, 4.13] | 69 | Not applicable | After omission, MD increased and confidence interval does not contain 0, P<0.00001, significant. Heterogeneity decreased. This study is the main reason for the non-significant primary effect. |
| Common Peroneal Nerve SCV | 3 | MD = 3.61 [-0.14, 7.35] | 99 | liu2015 | MD = 3.13 [-2.33, 8.60] | 99 | Not applicable | After omission, the MD confidence interval contains 0, P=0.26, not statistically significant. This study has an important influence on the effect significance. |
| Common Peroneal Nerve SCV | 3 | MD = 3.61 [-0.14, 7.35] | 99 | yao2016 | MD = 5.00 [1.43, 8.58] | 98 | Not applicable | After omission, MD increased and confidence interval does not contain 0, P=0.006, significant. This study is the key reason the primary effect was near the threshold. |
| Common Peroneal Nerve SCV | 3 | MD = 3.61 [-0.14, 7.35] | 99 | zhang2018 | MD = 2.05 [-1.26, 5.36] | 97 | Not applicable | After omission, MD decreased, confidence interval contains 0, P=0.23, not statistically significant. This study has a major influence on the pooled effect. |
| Common Peroneal Nerve SCV | 3 | MD = 3.61 [-0.14, 7.35] | 99 | zu2020 | MD = 4.24 [-0.51, 8.99] | 99 | Not applicable | After omission, MD increased slightly, confidence interval is close to 0, P=0.08, threshold. This study has a certain influence on the effect significance. |

**Abbreviations:** CI, confidence interval; I², I-squared statistic (heterogeneity); MD, mean difference; MNCV, motor nerve conduction velocity; SCV, sensory conduction velocity; SMD, standardized mean difference; SNCV, sensory nerve conduction velocity.

**Note:** "P for Subgroup Difference" is listed as "Not applicable" because the leave-one-out sensitivity analysis does not involve between-subgroup comparisons. All original data points (numerical values, brackets, punctuation) from the source document have been preserved without alteration.

# 7 Supplementary Material S7 . Assessment of evidence quality for each outcome

| **Quality assessment** | | | | | | | | | | | | | | | | | **No of patients** | | | | | | | **Effect** | | | | | | **Quality** | **Importance** |
| --- | --- | --- | --- | --- | --- | --- | --- | --- | --- | --- | --- | --- | --- | --- | --- | --- | --- | --- | --- | --- | --- | --- | --- | --- | --- | --- | --- | --- | --- | --- | --- |
|  |  |  |  |  |  |  |  |  |  |  |  |  |  |  |  |  |  |  |  |  |  |  |  |  |  |  |  |  |  |  |  |
| **No of studies** | **Design** | | | | | **Risk of bias** | **Inconsistency** | | | **Indirectness** | | | | **Imprecision** | | **Other considerations** | **Treatment** | | | **Control** | | | | **Relative (95% CI)** | | **Absolute** | | | |  |  |
| **Overall Effective Rate (Dichotomous; Better indicated by higher values)** | | | | | | | | | | | | | | | | | | | | | | | | | | | | | | | |
| 10 | RCTs | | | | | serious^1^ | not serious^a^ | | | not serious | | | | not serious^b^ | | strong association, reporting bias^4^ | 394 / 426 | | | 334 / 424 | | | | RR 1.18 (1.12 to 1.25) | | — | | | | ⬇⬇⬇◯ MODERATE | CRITICAL |
| **Total TCM Syndrome Score (Continuous; Better indicated by lower values)** | | | | | | | | | | | | | | | | | | | | | | | | | | | | | | | |
| 4 | RCTs | | | | | serious^1^ | not serious^a^ | | | not serious | | | | serious^3^ | | none | 172 | | | 172 | | | | — | | SMD -0.76 (-0.98 to -0.54) | | | ⬇⬇◯◯ LOW | | CRITICAL |
| **Motor Nerve Conduction Velocity - Composite MCV (Continuous; Better indicated by higher values)** | | | | | | | | | | | | | | | | | | | | | | | | | | | | | | | |
| 11 | RCTs | | | | | serious^1^ | very serious^2^ | | | not serious | | | | not serious^b^ | | none | ~468 | | | ~458 | | | | — | | SMD 1.85 (1.08 to 2.62)^c^ | | | | ⬇◯◯◯ VERY LOW | CRITICAL |
| **Sensory Nerve Conduction Velocity - Composite SCV (Continuous; Better indicated by higher values)** | | | | | | | | | | | | | | | | | | | | | | | | | | | | | | | |
| 11 | RCTs | | | | | serious^1^ | very serious^2^ | | not serious | | | | | not serious^b^ | | none | ~468 | | | | ~458 | | | — | | SMD 1.46 (0.86 to 2.06)^c^ | | | | ⬇◯◯◯ VERY LOW | CRITICAL |
| **Common Peroneal Nerve MCV (Continuous; Better indicated by higher values)** | | | | | | | | | | | | | | | | | | | | | | | | | | | | | | | |
| 9 | RCTs | | | | serious^1^ | | very serious^2^ | | not serious | | | | | not serious^b^ | | none | ~398 | | | | ~398 | | | — | | SMD 1.01 (0.48 to 1.54) | | | ⬇◯◯◯ VERY LOW | | IMPORTANT |
| **Oxidative Stress Marker: SOD (Continuous; Better indicated by higher values)** | | | | | | | | | | | | | | | | | | | | | | | | | | | | | | | |
| 2 | RCTs | | | serious^1^ | | | not serious^a^ | not serious | | | | | | serious^3^ | | none | | 82 | | | | 82 | | — | | MD 12.34 U/mL higher (5.21 to 19.47 higher)^d^ | | ⬇⬇◯◯ LOW | | | IMPORTANT |
| **Oxidative Stress Marker: MDA (Continuous; Better indicated by lower values)** | | | | | | | | | | | | | | | | | | | | | | | | | | | | | | | |
| 2 | RCTs | | serious^1^ | | | | not serious^a^ | | not serious | | | | | serious^3^ | | none | | 82 | | | | | 82 | — | | MD -1.32 μmol/L lower (-2.15 to -0.49 lower)^d^ | | ⬇⬇◯◯ LOW | | | IMPORTANT |
| **Toronto Clinical Scoring System (TCSS) Score (Continuous; Better indicated by lower values)** | | | | | | | | | | | | | | | | | | | | | | | | | | | | | | | |
| 2 | RCTs | | serious^1^ | | | | not serious^a^ | | | | not serious | | | serious^3^ | none | | | 97 | | | | | 97 | | — | | MD -2.65 (-3.88 to -1.42) | ⬇⬇◯◯ LOW | | | IMPORTANT |
| **Glycated Hemoglobin (HbA1c, %) (Continuous; Better indicated by lower values)** | | | | | | | | | | | | | | | | | | | | | | | | | | | | | | | |
| 2 | RCTs | | serious^1^ | | | | not serious^a^ | | | | | not serious | serious^3^ | | none | | | 102 | | | | | 102 | | — | | MD -0.36 (-0.67 to -0.05) | ⬇⬇◯◯ LOW | | | IMPORTANT |
| **Adverse event rate (Better indicated by lower values)** | | | | | | | | | | | | | | | | | | | | | | | | | | | | | | | |
| 3 | RCTs | serious^1^ | | | | | not serious^a^ | | | | | not serious | | very serious^3^ | | none | | | 12 / 157 | | | | 13 / 156 | | RR 0.91 (0.43 to 1.92) | | — | ⬇◯◯◯ VERY LOW | | | IMPORTANT |

**Explanations for downgrading the quality of evidence (as per your analysis):**

^1^ **Risk of Bias (serious):** Majority of included RCTs had unclear or high risk of bias regarding random sequence generation, allocation concealment, and blinding (performance & detection bias), as assessed by the Cochrane RoB tool.

^2^ **Inconsistency (very serious for NCV outcomes):** Extreme and unexplained heterogeneity was observed in all nerve conduction velocity outcomes (I² > 90-96%), as detailed in the five-tiered analytical framework. The pooled estimates are considered unreliable.

^a^ **Inconsistency (not serious):** For outcomes like effective rate and TCM scores, heterogeneity was low (I² < 50%) and point estimates were consistent.

^3^ **Imprecision (serious/very serious):** Downgraded due to limited number of studies (often < 5) and/or total sample size (< 400) leading to wide confidence intervals that include both appreciable benefit and no effect.

^b^ **Imprecision (not serious):** Not downgraded for NCV outcomes due to large total sample size (~900), despite wide CIs stemming from high heterogeneity.

^4^ **Other Considerations - Reporting Bias (suspected):** Funnel plot asymmetry and Egger's test suggested potential publication bias for the overall effective rate outcome.

^c^ **Effect Note for NCV:** The pooled SMD for composite MCV/SCV is presented statistically but should be interpreted with extreme caution due to fundamental heterogeneity. The confidence interval is extremely wide.

^d^ **Effect Note for Oxidative Stress:** Mean Difference (MD) values are illustrative approximations based on data from Song 2017 and Zu 2020. Please verify and insert the precise pooled MD from your meta-analysis.

**General Notes & Abbreviations:**

- This table is generated based on the 12 RCTs (N=926) included in your systematic review of Buyang Huanwu Decoction + ALA for DPN.
- **RCT:** Randomized Controlled Trial; **SMD:** Standardized Mean Difference; **MD:** Mean Difference; **RR:** Risk Ratio; **CI:** Confidence Interval.
- **Patient Numbers:** Values for intervention and control groups are approximate totals from your included studies. Specific numbers for some outcomes (e.g., NCV) are estimates based on the studies reporting that outcome.
- **GRADE Quality Symbols Legend:** ⬇⬇⬇⬇ = HIGH; ⬇⬇⬇◯ = MODERATE; ⬇⬇◯◯ = LOW; ⬇◯◯◯ = VERY LOW.
- **Importance:** Judged as per your review's objectives. "CRITICAL" for decision-making (e.g., efficacy, safety), "IMPORTANT" for supportive outcomes.

**Action Required:** Please **replace the illustrative effect estimates (SMD/MD/RR with 95% CI)** in the "Effect" columns with the **exact pooled results** from your meta-analysis performed in RevMan or other software. The current values are placeholders based on the description in your manuscript.
